# Immunity beyond borders: non-lethal *Plasmodium* confers cross-protection against lethal *Babesia* via macrophage activation

**DOI:** 10.3389/fimmu.2026.1805513

**Published:** 2026-05-05

**Authors:** Iqra Zafar, Yongchang Li, Daisuke Kondoh, Shimaa Abd El-Salam El-Sayed, Tanjila Hasan, Tomoyo Taniguchi, Hang Li, Noboru Inoue, Kentaro Kato, Xuenan Xuan

**Affiliations:** 1 National Research Centre for Protozoan Diseases, Obihiro University of Agriculture and Veterinary Medicine, Obihiro, Hokkaido, Japan; 2 Laboratory of Sustainable Animal Environment, Graduate School of Agricultural Science, Tohoku University, Osaki, Miyagi, Japan; 3 College of Veterinary Medicine, Xinjiang Agricultural University, Ürümqi, China; 4 Department of Veterinary Medicine, Obihiro University of Agriculture Veterinary Medicine, Obihiro, Hokkaido, Japan; 5 Department of Biochemistry and Chemistry of Nutrition, Faculty of Veterinary Medicine, Mansoura University, Mansoura, Dakahlia, Egypt; 6 Department of Medicine and Surgery, Faculty of Veterinary Medicine, Chattogram Veterinary and Animal Sciences University, Chattogram, Bangladesh; 7 Department of Immunology and Parasitology, Graduate School of Medicine, University of the Ryukyus, Nishihara Cho, Okinawa, Japan; 8 School of Life and Health Sciences, Hainan University, Haikou, China; 9 Research Center for Asian Infectious Disease, The Institute of Medical Science, The University of Tokyo, Minato, Tokyo, Japan

**Keywords:** *Babesia rodhaini*, babesiosis, co-infection, cross-protection, innate immunity, macrophages, malaria, *Plasmodium berghei* XAT

## Abstract

**Introduction:**

In co-endemic regions, hosts are concurrently infected by related apicomplexan hemoparasites, *Plasmodium* and the zoonotic pathogen *Babesia*. This study investigates how *Plasmodium berghei* XAT-*Babesia rodhaini* co-infection modulates host immunity, dissecting the contributions of innate and adaptive immune cells.

**Methods:**

Disease progression was assessed by survival, parasitemia, body weight, and hematological parameters. Splenic histopathology and qPCR were used to evaluate tissue damage and parasite burden. Immune responses were analyzed by cytokine, antibody measurement, and flow cytometry. Adaptive immunity was examined using SCID mice, and the roles of innate effectors were determined through selective depletion of NK cells and macrophages.

**Results:**

*P*. *berghei* XAT infection elicited robust, heterologous protection significantly reducing parasitemia and splenic damage. This was linked with potent pro-inflammatory cytokines (IFN-γ, IL-12p70, and TNF-α), balanced by increased IL-10 levels, alongside increased ROS/NO production. While co-infected SCID mice, and NK depletion retained protection, macrophage depletion eliminated cross-protection and altered microbicidal cytokine and ROS/NO profiles. Flow cytometry confirmed expansion of the splenic macrophages in protected mice.

**Conclusion:**

These findings demonstrate that cross-protection is mediated by a macrophage-driven innate response. Protection was associated with IFN-γ-driven pro-inflammatory responses and ROS/NO production, balanced by IL-10 to limit immunopathology. This “protective homeostasis” highlights macrophage-targeted immunomodulation as a promising strategy for multivalent vaccines against apicomplexan parasites. Future research is warranted to elucidate macrophage activation and polarization dynamics underlying this protection.

## Introduction

In natural settings, hosts are rarely infected by a single pathogen. Instead, frequent exposure to multiple infectious agents results in dynamic co-infections that profoundly shape disease progression (1, 2). Consequently, clinical outcomes are governed by a complex interplay of factors, including the sequence of infection and inter-pathogen competition, which modulate the host immune response (3, 4). This paradigm is particularly pertinent to apicomplexan parasites such as *Plasmodium* and *Babesia*, which often share overlapping ecological niches and vectors. The first documented human co-infection with both parasites was reported in 1983 (5). Since then, numerous cases have been identified across various geographic regions (6–9). However, these reports have primarily focused on documenting the occurrence of co-infections. They have not addressed critical aspects such as disease severity, clinical outcomes, or the underlying immunological mechanisms governing these complex parasite-host interactions. As phylogenetically related, intraerythrocytic hemoparasites, both *Plasmodium* and *Babesia* impose a substantial global health burden. They cause significant morbidity and mortality in both human and veterinary populations (10–13).

While malaria caused by *Plasmodium* spp. has long been recognized as a major global health burden, babesiosis has historically been viewed primarily as a veterinary disease. However, over the past two to three decades, human babesiosis has emerged as a serious public health concern. It is now recognized as an emerging zoonosis that particularly affects immunocompromised individuals (13, 14). Despite its massive economic impact on the livestock industry and its rising threat to human health, our understanding of *Babesia* co-infections, pathogenesis, and immunity remains relatively sparse (6, 7). Understanding these interactions could reveal fundamental principles of heterologous immunity, where exposure to one pathogen confers protection against another. This phenomenon has profound implications for rational vaccine design (15).

The principle of heterologous immunity is historically founded on Edward Jenner’s seminal use of cowpox to confer protection against smallpox. This remains a landmark demonstration of cross-species protective immunity (16). This phenomenon has been well documented among various *Plasmodium* species and strains. A primary example is the protection conferred by *Plasmodium berghei* XAT against otherwise lethal *P*. *berghei* NK65 infections. Although early studies did not elucidate the underlying mechanisms (17), subsequent work suggested an association with the expansion of B220^int^ CD11c^+^ cells. Enhanced IL-10 mRNA expression following infection with non-lethal *P. berghei* XAT or *P. yoelii* 17X may also be involved (18). Beyond intra-species interactions, research has also identified cross-protection between distinct genera of blood-borne parasites. Studies in mice and macaques have shown that *Babesia microti* protects against subsequent *Plasmodium* infections, though the mechanism was initially unclear (19, 20). Later studies correlated this protection to the activation of the innate immune system. This includes the surge of pro-inflammatory monocytes during *P. cynomolgi* infection (21). Recently, this protective effect has been attributed to macrophage activity (22). These observations underscore the capacity of antigenically distinct yet related parasites to elicit cross-protective immune responses. Together, these findings raise critical questions regarding the immune mechanisms and the relative contributions of innate and adaptive immune responses. Both innate and adaptive components, including macrophages, dendritic cells, and lymphocytes, are putative mediators of cross-protection. However, the specific contribution of each remains incompletely defined (23–26). Notably, antibody-mediated immunity is not universally required for protection against hemoparasitic infections. Effective cross-protection has been reported even with reduced or delayed antibody responses (13, 22). Collectively, these findings highlight a prominent and potentially dominant role for innate immune mechanisms in shaping heterologous immunity.

The close phylogenetic relationship between *Babesia* and *Plasmodium* suggests the potential for shared, exploitable immune pathways (27, 28). In support of this, we previously demonstrated that primary *Babesia microti* infection confers cross-protective immunity against a subsequent *Plasmodium* challenge (29). Yet, the inverse relationship, whether *Plasmodium* infection elicits protection against *Babesia*, remains unresolved. Elucidating this interaction is necessary for identifying broad-spectrum immunomodulatory targets requisite for rational design of multivalent vaccines. Such a strategy would offer significant cross-protective efficacy alongside substantial economic and biomanufacturing advantages.

In this study, we investigated the protective capacity of *Plasmodium berghei* XAT against lethal *Babesia rodhaini*. Specifically, we examined the relative contributions of innate versus adaptive effector populations in mediating this heterologous defense. We hypothesized that primary exposure to non-lethal *Plasmodium* induces a state of trained innate immunity that restricts subsequent *Babesia* proliferation independently of B and T cell involvement.

## Materials and methods

### Experimental animals

For this study, 6–8 weeks old female wild-type BALB/c mice were obtained from CLEA Japan. The animals were maintained under pathogen-free conditions as described by Li et al. (30). Female mice were included to account for sex as a biological variable, as immune responses differ significantly between males and females (31). Females often exhibit more robust immune responses and, despite hormonal cycling, are not inherently more variable than males (32–34). This choice also avoids the immunosuppressive effects of testosterone, which can delay macrophage activation and impair adaptive immunity. Using females, therefore, reduces hormonal confounding and provides a more stable model for studying host-parasite interactions (35, 36).

### Maintenance of parasite and study design

Cryopreserved stabilates of *Babesia rodhaini* (Australia strain) were taken from the cell bank, and *Plasmodium berghei* XAT was supplied by Nagasaki University, with assistance partially funded by the National BioResource Project (NBRP), MEXT, Japan. After thawing, the stabilates were passaged in mice via intraperitoneal injection and maintained for subsequent experiments. The study aimed to evaluate the impact of a primary, non-lethal *P. berghei* XAT infection on a subsequent lethal *B. rodhaini* challenge. Mice were divided into three test groups (n = 6 each) that were intraperitoneally (i.p.) inoculated with 10^5^ P*. berghei* XAT-infected red blood cells (iRBCs). These groups were then challenged with 10^3^ *B. rodhaini*-infected RBCs on days 14, 28, 70, and 100 post-primary infection (referred to as co-infected or PbXAT/Br14, PbXAT/Br28, PbXAT/Br70, and PbXAT/Br100, respectively). A positive control group (Br, n = 6) received only 10^3^ *B. rodhaini*-infected RBCs, while another group (PbXAT, n = 6) was infected solely with *P. berghei* XAT iRBCs. The experimental groups included a Naïve group injected with PBS. Collectively, these groups were used to examine the effects of co-infection.

In the initial trial, six groups (PbXAT, PbXAT/Br14, PbXAT/Br28, PbXAT/Br70, Br, and Naïve) were monitored to assess parasitemia progression, body weight, blood parameters, and other physiological markers. A second trial included four groups (Br, PbXAT/Br14, and Naïve, n = 5 each) to quantify parasite load in blood and spleen, and to investigate the host immune response. This included analyses of spleen immune cell populations, histopathology, antibody levels, and cytokine responses. A subsequent trial was conducted with the PbXAT/Br14 group, where challenge infections were performed using a lysate of *P. berghei* XAT instead of live parasites. This trial also included *B. rodhaini* and Naïve groups (n = 5) to explore the role of dead parasites in eliciting immunity. Finally, the mice were again infected, following the outlined design to perform histopathology.

All procedures were performed under general anesthesia. Induction was achieved using 4-5% isoflurane (Viatris Healthcare G.K., Tokyo, Japan) delivered in 100% oxygen at a flow rate of 1 L/min via a Forane vaporizer (Forawick; Muraco Medical Co., Ltd., Tokyo, Japan). Following induction, anesthesia was maintained with 1.5-2.5% isoflurane administered through a nose cone. At the end of the experiment, mice were euthanized by isoflurane overdose (≥4-5%), followed by cervical dislocation as a secondary method to ensure death (37, 38).

### Monitoring survival, parasitemia, and hematological parameters in mice

Mice were regularly monitored to assess survival rates, parasitemia, body weights, and hematological parameters. Red blood cell (RBC) counts and hematocrit (HCT) values were measured using the Celltac Alpha MEK-6550K analyzer (Nihon Kohden) (25). Parasitemia was checked every other day by preparing thin blood smears stained with Giemsa, followed by microscopic examination of 10³ infected red blood cells (iRBCs) under a 100× oil immersion lens using the Eclipse E200 microscope (Nikon, Tokyo, Japan). The use of blood smears to quantify parasitemia made it challenging to distinguish between *Plasmodium* and *Babesia* parasite levels in co-infected mice. Therefore, the parasitemia levels were reported as combined values to provide an overall representation.

### Quantification of parasite burden by qPCR

At day 7 post-challenge infection (pci), spleen was harvested, and blood was collected from mouse groups (Br, PbXAT, and PbXAT14/Br; n=5). DNA was extracted from tissues and blood using NucleoSpin^®^ Tissue Kit (Takara, Japan), and QIAamp^®^ DNA Blood Mini Kit (Qiagen, Hilden, Germany), respectively, adhering to the directions specified by the manufacturer. The DNA was eluted to a final volume of 100 μL. To quantify the parasites, a pair of specific primers (F: 5`-AAGCATTAAATAAAGCGAATACATCCTTAC-3`; R: 5`GGAGATTGGTTTTGACGTTTATGTG-) was used to amplify a part of the Pb 18S rRNA gene (134 bp) (39, 40). Quantification of *B. rodhaini* was carried out using a newly designed primer pair specific to the β-tubulin gene sequence (F: 5’-CCAGGTCATTGATAACGAAGC-3’; R: 5’-TAACACCACTCATAGCGGCA-3’), which amplifies a 113 bp fragment. A final volume reaction of 10 μL was run in duplicates, composed of 5 μL 1 × PowerUp™ SYBR™ Green Master Mix (Applied Biosystems, Massachusetts, USA), 0.8 μM primers, 1 uL DNA, and 2.4 uL UltraPure™ DNase/RNase-free water (Thermo Fisher Scientific). The PCR conditions for *P*. *berghei* XAT were: 50 °C for 2 min, 95 °C for 2 min, 40 cycles of 95 °C for 15 sec and 60 °C for 45 sec, with a dissociation stage. Conditions for *B*. *rodhaini* were: 50 °C for 2 minutes, 95 °C for 2 minutes, followed by 40 cycles of 95 °C for 15 seconds and 60 °C for 1 minute, with a final dissociation step. The reactions were run in the QuantStudio™ 5 Real-time PCR System (Thermo Fisher Scientific). Serially diluted standards prepared from plasmids served as reference samples. Standard curves were generated from serial dilutions of plasmid DNA to enable the quantification of parasites based on the mean quantification cycle (Cq) values from duplicate samples. Parasite counts were log-transformed before statistical analysis.

### Preparation of inactivated *P*. *berghei* XAT for mouse immunization

To evaluate whether immunization with dead *P. berghei* XAT protects against *B. rodhaini* infection, mice were repeatedly vaccinated with glutaraldehyde-fixed *P. berghei* XAT-parasitized red blood cells (pRBCs) or non-parasitized red blood cells (npRBCs) as previously described (41) (25). In brief, we collected *B*. *microti*-infected red blood cells (RBCs) from mice when parasitemia was more than 50%. After removal of the plasma and buffy coat, the RBCs were washed three times in sterile PBS (pH 7.2). Following the final wash, the cells were fixed in 0.25% glutaraldehyde for 15 minutes at room temperature. After three more washes in sterile PBS supplemented with penicillin and streptomycin, the fixed RBCs were stored at 4 °C in sterile 5% dextrose with antibiotics until further use. The test groups (n=5) received three immunizations, each 14 days apart, with 10^8^ glutaraldehyde-fixed *P. berghei* XAT-pRBCs, while the naïve mice were administered glutaraldehyde-fixed npRBCs. One week following the final immunization, blood samples were collected from all mice and analyzed using an enzyme-linked immunosorbent assay (ELISA) to assess the specific humoral response against dead *P. berghei* XAT. Subsequently, both groups were challenged with *B. rodhaini* to check the effect of immunizations with glutaraldehyde-fixed pRBCs and npRBC.

### Preparation of *P*. *berghei* XAT lysate

The preparation of *P. berghei* XAT crude antigen was carried out using a previously described protocol (42). Briefly, intracardiac blood samples were collected from mice when parasitemia levels exceeded 30%. The blood was centrifuged at 3,000 × g for 5 minutes, and the pelleted red blood cells (RBCs) were chemically lysed with an equal volume of 0.15% saponin in PBS for 5 minutes at room temperature. The suspension was then centrifuged, and the parasite pellets were washed three times with 1× PBS before re-suspending in PBS. For mechanical disruption, the parasites underwent two sonication cycles for 30 seconds each at 40% amplitude. The protein concentration of the resulting lysate was determined using the Pierce™ BCA Protein Assay Kit (Thermo Fisher Scientific, USA). The lysate was stored at −20 °C for subsequent use as a coating reagent for ELISA plates.

### Flow cytometric analyses of mouse splenocytes

**Figure 1:**
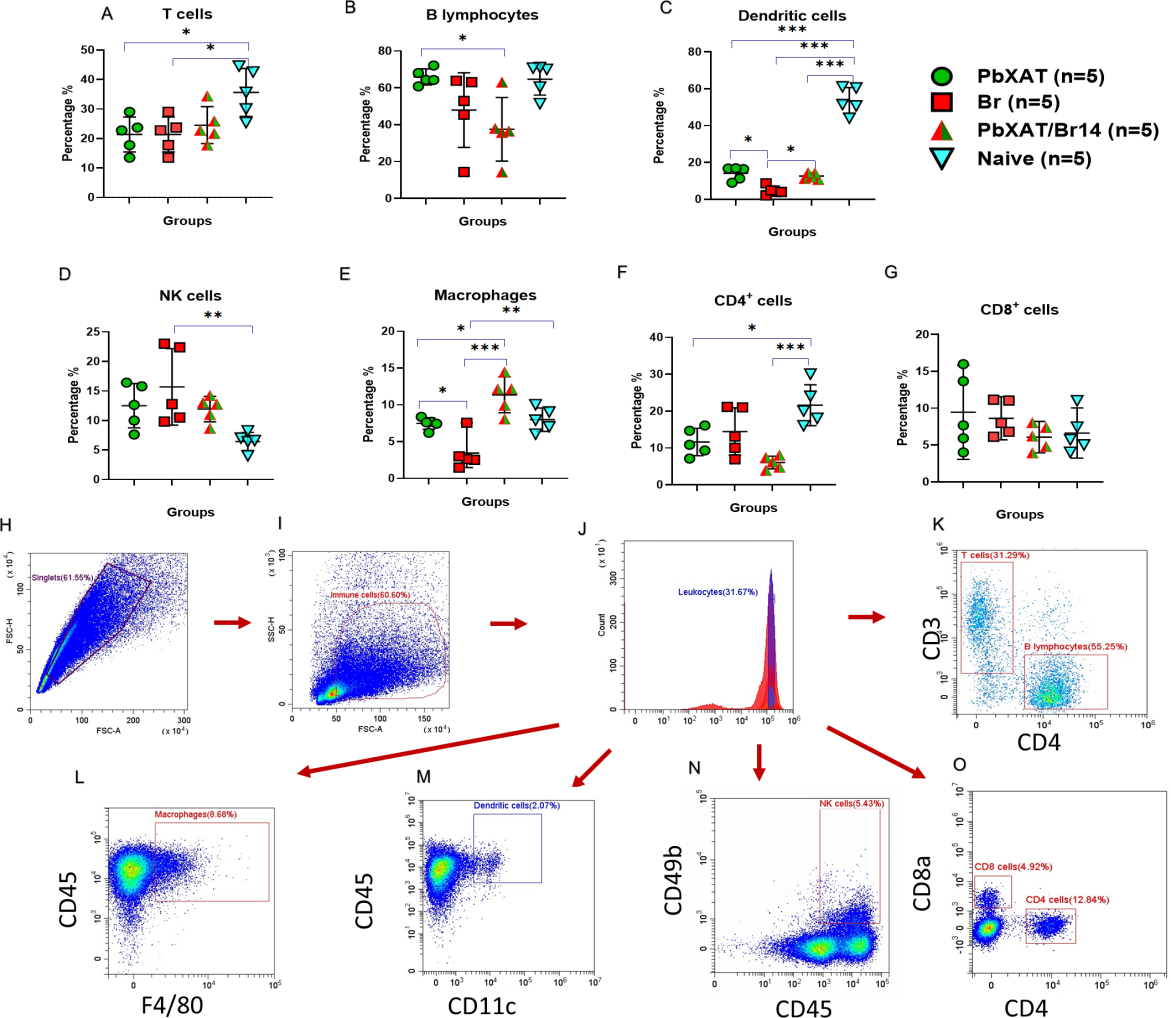
Immunophenotyping of different types of immune cells in the spleen from experimental groups (n=5) at day 7 pci. **(A)** CD45^+^ CD3^+^ cells (T cells), **(B)** CD45^+^ CD19^+^ cells (B lymphocytes), **(C)** CD45^+^ CD11c+ cells (Dendritic cells), **(D)** CD45^+^ CD49b^+^ cells (Natural killer cells), **(E)** CD45^+^ F4/80^+^ cells (Macrophages), **(F)** CD45^+^ CD4^+^, **(G)** CD45^+^ CD8^+^. Data represent the mean ± SD from a single experiment (n = 5 mice per group). Analysis of variance (ANOVA) was followed by *post hoc* Tukey’s multiple comparison test. Asterisks indicate statistical significance (*p < 0.05; **p < 0.01, and ***p < 0.001). The percentage population of each cell type is presented as mean ± SD. Gating scheme for fluorescence-activated sorting (FACS) analysis of immune cells. A total of 50,000 events were analyzed. Each panel is a representative image of gating for **(H)** singlets, **(I)** CD45^+^ (immune cells), **(J)** CD45^+^ leukocytes, **(K)** CD45^+^ CD3^+^ (T cells), CD19^+^ (B cells), **(L)** CD45^+^ F4/80^+^ (macrophages), **(M)** CD45^+^ CD11c^+^ (dendritic cells), **(N)** CD45^+^ CD49b^+^ (natural killer cells), and **(O)** CD45^+^ CD8a^+^ (CD8 T cells), CD45^+^ CD4^+^ (CD4 T cells).

Spleens were aseptically removed from all mouse groups (n=5) at day 7 pci. Single-cell suspensions were prepared by mincing spleen tissue into small fragments and filtering them through a sterile 70 µm cell strainer into a 50 mL tube to ensure a homogeneous splenocyte suspension. Cell suspensions were washed twice with ice-cold 1× PBS by centrifugation at 375 × g for 5 min at 4 °C. The pellets were subsequently treated with 1× ACK lysis buffer (Gibco, Massachusetts, USA) for 5 min at room temperature to remove residual erythrocytes. The lysis reaction was halted by adding cold PBS, and the samples were centrifuged again under the same conditions. The final cell pellets were resuspended in 2 mL of cell staining buffer (BioLegend, California, USA) and stored on ice until further staining. Trypan blue staining was performed (1:5 dilution), and viable cell counts were determined by a hemocytometer. For each sample, approximately 1 × 10^6^ splenocytes were resuspended in cell staining buffer (CSB) and pelleted by centrifugation. The cells were then incubated with 70 µL of CSB containing CD16/CD32 monoclonal antibody (Invitrogen, Massachusetts, USA) to block Fc receptors for 25 minutes at 4 °C. Following this step, the cells were stained with marker-specific antibodies conjugated to fluorophores for 30 minutes at 4 °C in the dark. Details of the antibody panel used are provided in **Supplementary Table 1.1**, **1.2**. The cells were then fixed in 200 µL of 4% paraformaldehyde (PFA) for 15 mins, followed by two washes with CSB and centrifugation at 375 × g for 5 min at 4 °C. In the final step, stained cells were resuspended in 200 µL of CSB and analyzed using a CytoFLEX flow cytometer (Beckman Coulter, California, USA). Data were processed with CytExpert 2.4 software (Beckman Coulter). The gating strategy employed was adapted from Bayne & Vonderheide (43), with the representative immunophenotyping strategy shown in **Figures 1H–O**.

### Histopathology and splenomegaly analyses

To evaluate the impact of infection on splenic architecture, mice from each experimental group (n = 5) were euthanized at day 7 pci. Spleens were harvested, weighed, and photographed to document the degree of splenomegaly. To account for variations in animal size, the spleen weight to body weight (SW/BW) ratio was calculated. For histopathological examination, samples collected from experimental groups were fixed in 4% paraformaldehyde, dehydrated through a series of graded alcohols, and embedded in paraffin. Tissue sections, 5 μm in thickness, were prepared, deparaffinized, and stained with hematoxylin and eosin (H&E). Tissue histology was analyzed by a histopathologist in a blinded manner using a Nikon Microphot-FX microscope, and images were captured with a Nikon Digital Sight DS-5M camera.

### Measurement of serum cytokine, ROS/RNS, and NO concentrations

Serum cytokine levels were analyzed in all mouse groups (PbXAT, Br, PbXAT/Br14, and Naïve, n=5) by collecting blood samples through cardiac puncture on day 7 post-primary infection or post-*B. rodhaini* infection. Serum was obtained by centrifuging the blood at 1,500 × g for 15 minutes at 4 °C. Serum concentrations of IFN-γ (catalog no. 88-7314-88), TNF-α (catalog no. 88-7324-88), IL-12p70 (catalog no. 88-7121-88), IL-6 (catalog no. 88-7064-88), IL-2 (catalog no. 88-7024-88), and IL-10 (catalog no. 88-7105-88) were quantified using Uncoated ELISA Kits (Thermo Fisher Scientific, Waltham, MA, USA) according to the manufacturer’s protocols. In addition, reactive oxygen and nitrogen species (ROS/RNS) levels were measured using the OxiSelect™ *In Vitro* ROS/RNS Assay (Cell Biolabs, Inc., catalog no. STA-347). For all assays, concentrations were calculated based on standard curves generated using known standards run in parallel on the same plate. Serum samples were diluted at a 1:100 ratio in PBS, and the optical density was measured using a MULTISKAN SkyHigh plate reader (Thermo Fisher Scientific), while fluorescence (480 nm excitation/530 nm emission) was read with the GloMax^®^-multi detection system (Promega, Wisconsin, USA).

### Assessment of humoral immune response

The humoral immune response was assessed by quantifying *P*. *berghei* XAT- and *B*. *rodhaini*-specific antibodies using ELISA. Serum samples were collected from the PbXAT, *B*. *rodhaini*, PbXAT/Br14, and Naïve groups (n=5) at day 7 pci. In co-infected mice, antibodies against both parasites were assessed. The antibody response was determined following our previous protocol (44). Nunc microtiter plates were coated with 50 µL of either *P*. *berghei* XAT lysate or recombinant GST-BrP26 antigen and incubated overnight at 4 °C. Following washing with PBST (PBS containing 0.05% Tween 20), plates were blocked with 3% skim milk in PBS for 1 hour at 37 °C. After another wash, 50 µL of serum samples (1:100 dilution in blocking buffer) were added and incubated for 1 hour at 37 °C. During incubation, HRP-conjugated goat anti-mouse immunoglobulin secondary antibodies (IgG, IgG1, and IgM) were prepared at a 1:4,000 dilution in a blocking solution. Following serum incubation, the plates were washed six times and incubated with the diluted secondary antibodies for 1 hour at 37 °C. After another round of washing, tetramethylbenzidine (TMB) substrate was added to each well and incubated for 1 hour at room temperature (RT) to detect antibody binding. The reaction was stopped by adding 2 M H_2_SO_4_ (25 μL/well). Absorbance readings were recorded at 415 nm using the MULTISKAN SkyHigh plate reader (Thermo Fisher Scientific). To ensure accuracy and reproducibility, all samples were analyzed in triplicate.

### Macrophages and NK cells depletion *in vivo*

To examine how NK cell and macrophage populations influence the observed cross-protective immunity, these immune cells were selectively depleted *in vivo*. First, NK cell depletion was achieved in BALB/c mice chronically infected with *P. berghei* XAT (day 26 pi). Mice were treated with 50 μl of anti-asialo GM-1 antibody (FUJIFILM Wako, Japan) diluted in 200 μl of PBS, administered i.p. on days −2, +3, and +6 relative to *B. rodhaini* infection. An equal volume of control rabbit antibody, diluted in 200 μl of PBS, was administered to control mice. Control mice received an equal dose of rabbit control antibody prepared in 200 μL of PBS.

**Figure 2:**
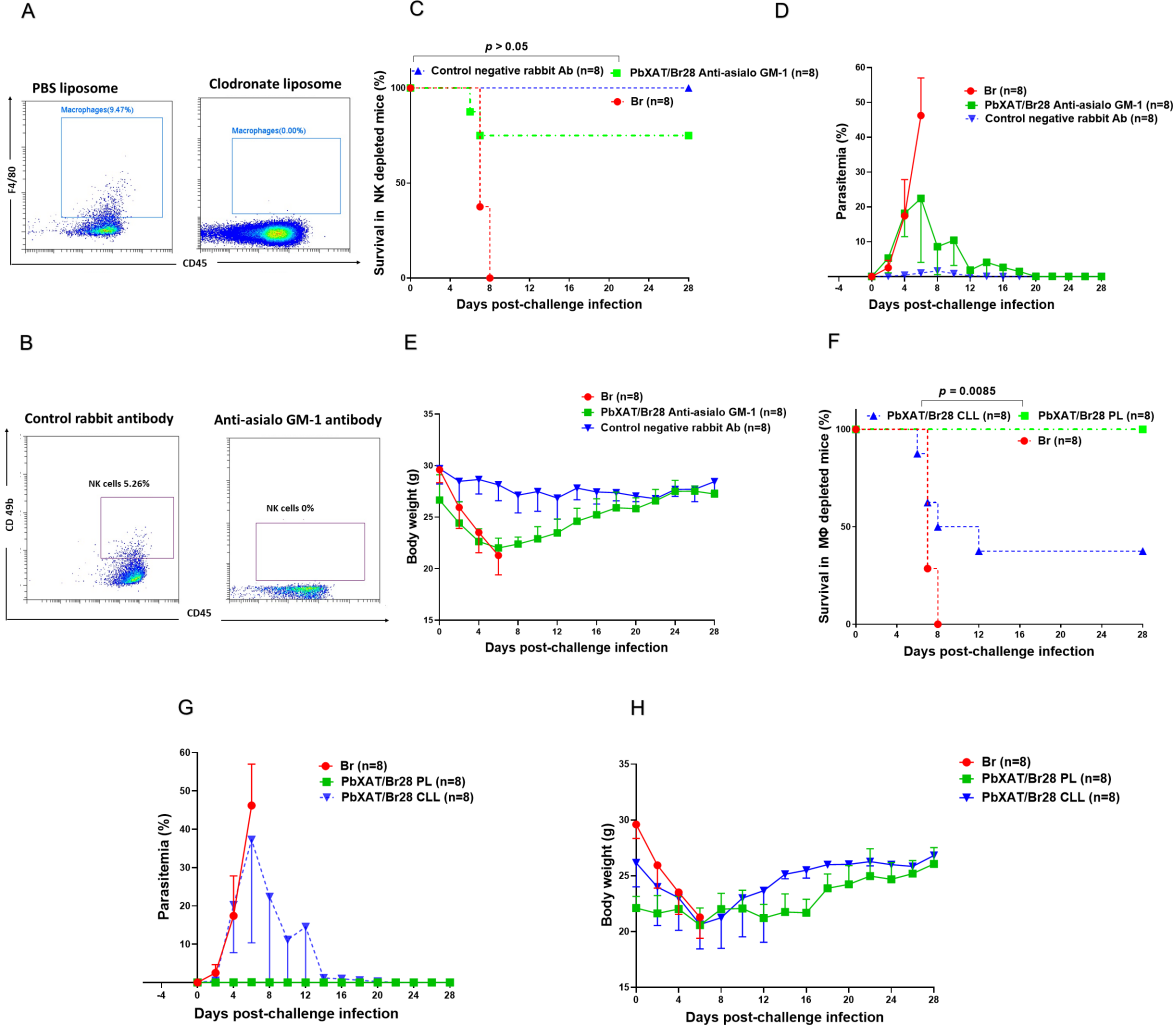
Efficiency of cellular depletion **(A, B)**. Representative flow cytometry plots demonstrating the depletion efficacy of **(A)** macrophages (F4/80^+^ CD45^+^) following PBS liposome control (PL) (left) compared to clodronate liposome (CLL) treatment (left), and **(B)** NK cells (CD49b^+^ CD45^+^) following control rabbit antibody administration (right) compared to anti-asialo GM-1 antibody (left). The role of NK cells in heterologous immunity is shown **(C–E)**. **(C)** Survival rates, **(D)** parasitemia, and **(E)** body weight changes in NK cell-depleted mice. Mice initially infected with *P*. *berghei* XAT and challenged with *B*. *rodhaini* (PbXAT/Br28) remained protected despite NK cell depletion (*p* > 0.05). The role of macrophages in heterologous immunity is shown **(F–H)**. **(F)** Survival rates, **(G)** parasitemia, and **(H)** body weight changes in macrophage-depleted mice. Depletion of macrophages via CLL significantly abolished the cross-protection in PbXAT28/Br mice (*p* = 0.0085), resulting in high parasitemia and increased mortality. Data represent mean ± standard deviation (SD) from eight mice per group (n = 8), and are representative of two independent experiments. Survival data were analyzed with the Kaplan-Meier non-parametric model. Group comparisons were performed using one-way ANOVA with Tukey’s *post hoc* test or a two-tailed Student’s t-test.

In a separate experiment, the contribution of macrophages to cross-protective immunity was examined by systemic macrophage depletion in chronically infected BALB/c mice (day 26 pi). Clodronate and PBS liposomes (CLL and PL, respectively) were purchased from www.liposoma.org (LIPOSOMA BV, Amsterdam, The Netherlands). Macrophages were depleted via intravenous injection of 300 µL clodronate liposomes. The first dose was administered 2 days before, and the second dose was given 3 days after challenge infection with *B*. *rodhaini*. The challenge infection was performed on day 28 following primary infection with *P*. *berghei* XAT. Control mice received 300 µL of PBS-containing liposomes. The efficacy of NK cell and macrophage depletion was confirmed one week after the last injection by staining splenocytes with antibodies against CD49b and F4/80, (Thermo Fisher Scientific, USA), respectively, and performing flow cytometry analysis (**Figures 2A, B**). The protocol for flow cytometry was the same as described in section 2.7, Flow Cytometric Analysis of Mouse Splenocytes. The above depletion protocols were adapted from previously published studies (22, 25, 45). Mice from depletion experiments (macrophage/NK cells) were used in two independent trials: the first was performed to monitor the course of infection and survival rates (n=8 per group), while the second was used to assess the antibody and cytokine levels at day 7 pci (n=4 per group).

### Statistical analyses

Statistical analyses were performed using GraphPad Prism version 8 (GraphPad Software, California, USA). Group comparisons were performed using ordinary one-way analysis of variance (ANOVA), followed by Tukey’s *post hoc* multiple-comparisons test, or a two-tailed unpaired Student’s t-test. Data are presented as mean values ± standard deviations. Survival analysis was done by using the log-rank and Wilcoxon tests, incorporating the Kaplan-Meier nonparametric model for establishing any statistically significant differences. A P-value of less than 0.05 was considered statistically significant, with results evaluated at a 95% confidence interval.

## Results

### Complete survival of mice initially infected with *P. berghei* XAT against lethal *B. rodhaini*

**Figure 3:**
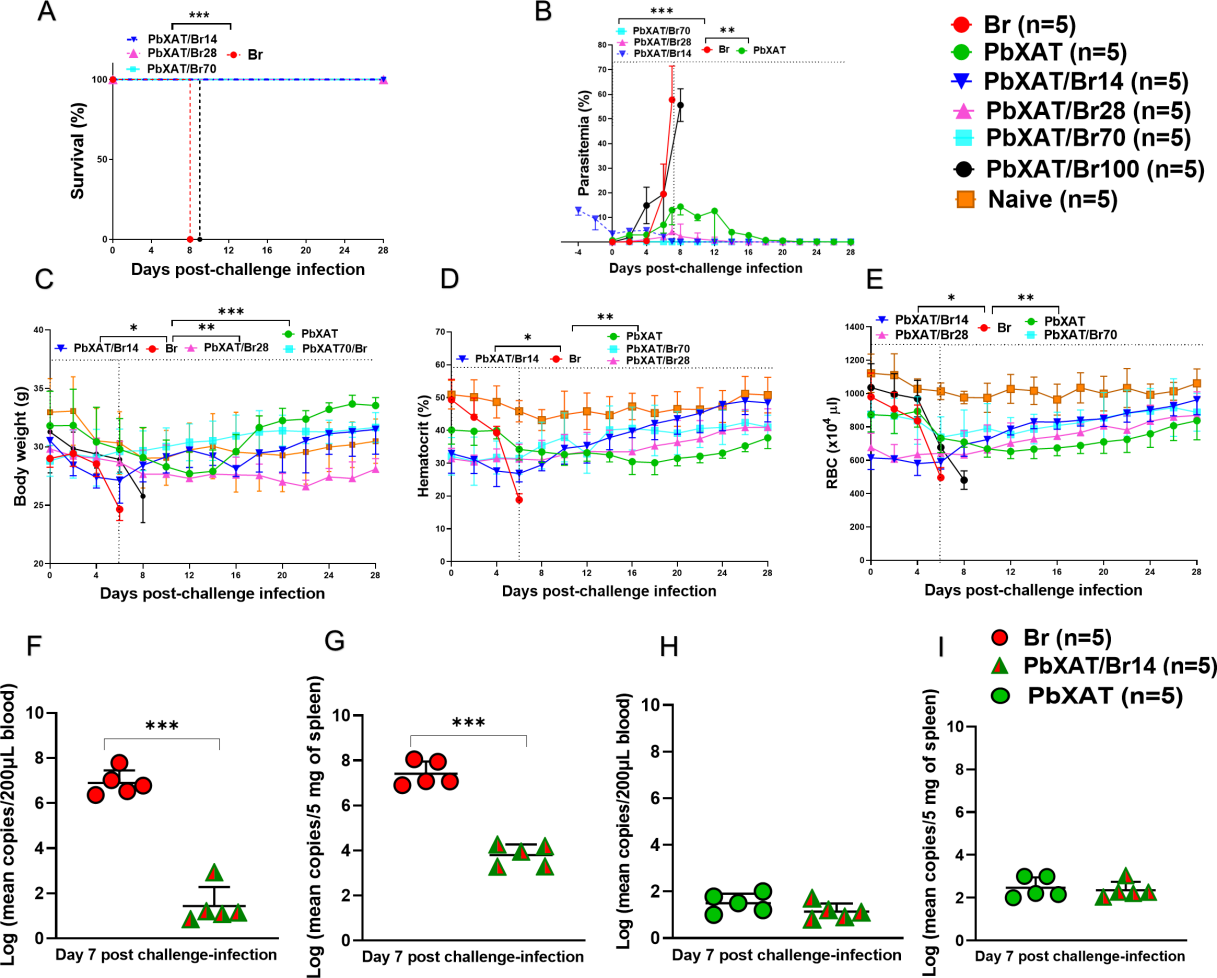
Disease progression and pathogen burden in all experimental groups. BALB/c mice were first infected with *P*. *berghei* XAT and subsequently challenged with *B*. *rodhaini* at days 14, 28, 70, or 100 post-primary infection. **(A)** Survival curve, **(B)** Parasitemia, **(C)** Body weight, **(D)** Hematocrit, **(E)** Red blood cell (RBC), values were monitored until death in Br and PbXAT/Br100 groups; all other groups (PbXAT/Br14, PbXAT/Br28, PbXAT/Br70, PbXAT, and Naïve) were observed for 28 days. Data represent mean ± standard deviation (SD) from five mice per group (n = 5), and are representative of two independent experiments. Survival data were analyzed with the Kaplan-Meier non-parametric model (*p* < 0.0001. Group comparisons were performed using one-way ANOVA with Tukey’s *post hoc* test or a two-tailed Student’s t-test. *B*. *rodhaini* and *P*. *berghei* XAT parasites were quantified by qPCR in **(F, H)** Blood and **(G, I)** Spleen tissue at day 7 post-challenge. Results are displayed as log−transformed mean copy numbers from mouse DNA (n=5 mice/group). Individual values are the means of duplicate samples. PbXAT/Br14 group showed reduced *B*. *rodhaini* sequestration compared to the Br group. Individual values are the means of duplicate samples. Log values were analyzed using a two-tailed unpaired Student’s t-test. Asterisks indicate statistical significance asterisks (*p < 0.05; **p < 0.01, and ***p < 0.001).

Infections involving *B*. *rodhaini* are characterized by aggressive parasitemia and significant lethality in murine models. To assess whether an initial infection with *P. berghei* XAT can confer protection against lethal *B. rodhaini* infection, BALB/c mice were first inoculated with *P. berghei* XAT and subsequently challenged with *B. rodhaini* at 14, 28, 70, and 100 days post-primary infection. The outcomes of *P. berghei* XAT primary infection varied among the mice. Mice infected only with *B. rodhaini* (Br) died by day 8 pci. In contrast, complete survival was observed in groups challenged on days 14 (PbXAT/Br14), 28 (PbXAT/Br28), and 70 (PbXAT/Br70) following the initial *P. berghei* XAT infection (**Figure 3A**). Survival was significantly improved in co-infected groups compared to Br mice (*p* < 0.0001, **Figure 3A**). However, mice challenged at 100 days post-primary infection (PbXAT/Br100) succumbed by day 9 pci. These findings indicate that *P. berghei* XAT offers full protection against lethal *B. rodhaini* infection up to 70 days post-primary infection. Beyond this timeframe, the cross-protective effect of *P. berghei* XAT diminishes and is no longer effective by day 100 (**Figure 3A**).

### Parasitemia and hematologic indices in *Plasmodium berghei* XAT and *Babesia rodhaini* co-infection

Parasitemia levels were assessed using Giemsa-stained blood smears, and the parasitemia progression for the three groups was represented in **Figure 3**. Parasitemia in *P*. *berghei* XAT-infected mice (referred to as PbXAT) was 14%. At day 0 pci, mice in the PbXAT/Br14 group exhibited a parasitemia level of 6.06%, attributed to the previous *P. berghei* XAT infection. Following the *B. rodhaini* challenge, parasitemia at day 6 pci in the PbXAT/Br14, PbXAT/Br28, and PbXAT/Br70 groups remained significantly lower than in the *B. rodhaini* (Br) single-infection group (*p* < 0.0001). The Br group reached a lethal peak parasitemia of 58% before death.

Notably, *P. berghei* XAT-infected red blood cells (pRBCs) were detectable in blood smears up to 26 days post-primary infection. Thereafter, parasitemia levels associated with *P. berghei* XAT declined to zero by day 28 pci (**Figure 3B**). Following challenge infection with *B. rodhaini* on day 28, parasitemia levels ranged from zero to low percentages (**Figure 3B**), reflecting a new wave of parasitemia induced by the *B. rodhaini* challenge. It is important to note that parasitemia observed in the PbXAT/Br14 group represents the combined contribution of *Plasmodium* and *Babesia* infections. In contrast to the PbXAT/Br14 group, no baseline parasitemia was detected in the PbXAT28 and PbXAT/Br70 groups, and observed parasitemia remained very low (**Figure 3B**).

Body weight analysis revealed transient weight loss in PbXAT and co-infected groups, which subsequently recovered as parasitemia resolved (**Figure 3C**). In Br mice, however, significant weight loss was observed, declining from an initial average of 29 g to 22.46 g before succumbing to infection. Co-infected mice experienced a gradual weight reduction followed by recovery after parasitemia subsided (**Figure 3C**). At day 6 pci, body weight in the PbXAT/Br14, PbXAT/Br28, and PbXAT/Br70 groups was significantly higher compared to the Br group (*p* = 0.04, *p* = 0.001, and *p* < 0.001, respectively).

All groups exhibited some degree of anemia, with Br mice showing severe reductions in red blood cell (RBC) counts and hematocrit levels. Anemia was evident in Br and PbXAT/Br100 mice shortly before death. Conversely, hematologic parameters in co-infected and PbXAT mice recovered and remained stable up to day 28 pci (**Figures 3D, E**). Hematocrit levels were significantly higher in the PbXAT/Br14 (*p* = 0.041), PbXAT/Br28 (*p* = 0.005), and PbXAT/Br70 (*p* = 0.009) than those in the Br group at day 6 pci. Similarly, RBC counts in these co-infected groups were significantly elevated compared to the Br group (*p* = 0.051, *p* = 0.031, and *p* = 0.008, respectively). The findings confirmed that increased parasitemia accelerated infected RBC (iRBC) breakdown, leading to reduced hematologic indices and anemia.

The findings confirmed that increased parasitemia accelerated infected RBC (iRBC) breakdown, leading to reduced hematologic indices and anemia. To exclude the possibility that the observed suppression of parasitemia in co-infected mice resulted from a failed establishment of infection due to inter-parasite competition, species-specific qPCR was performed. This approach allowed us to determine how much of the total parasitemia was attributable to *Babesia* versus *Plasmodium*. The results confirmed the presence of *B*. *rodhaini* DNA in both the blood (**Figure 3F**) and spleen (**Figure 3G**) of all co-infected mice. Notably, the *B*. *rodhaini* load was significantly lower in PbXAT/Br14 compared to the Br group in both blood and spleen (*p* < 0.001). In contrast, qPCR targeting *P*. *berghei* XAT showed a low parasite burden in both PbXAT and PbXAT/Br14 groups. This low parasite burden was expected, as the *P*. *berghei* XAT parasitemia was already in the resolution phase by day 21 post-primary infection (day 7 post-challenge). These data indicate that the cross-protection conferred by *P*. *berghei* XAT restricts *B*. *rodhaini* replication and sequestration rather than simply preventing the establishment of infection.

### Immunization with inactivated *P*. *berghei* XAT proves insufficient for conferring resistance against a fatal *B*. *rodhaini* challenge

**Figure 4:**
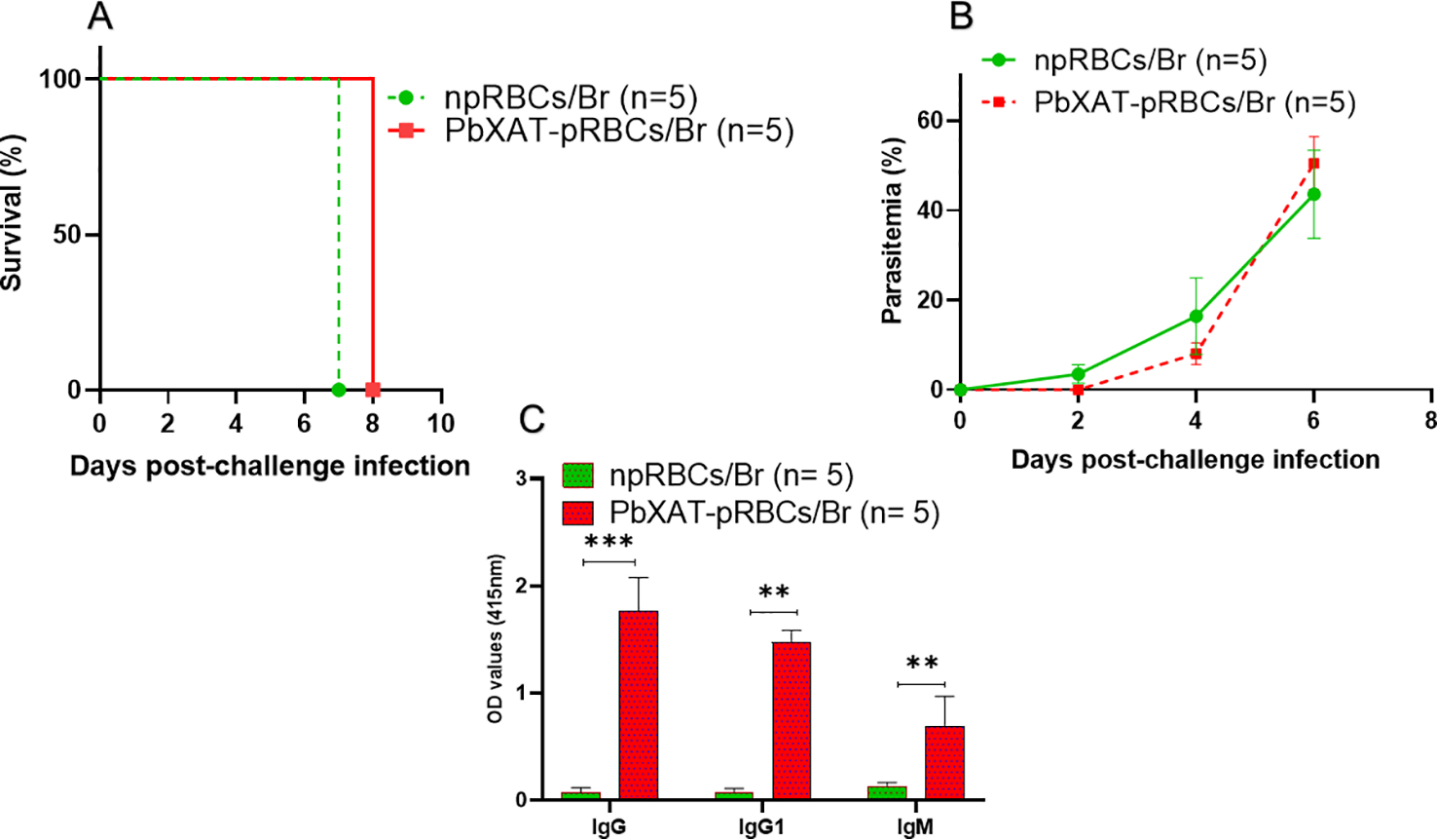
Evaluation of heterologous protection and humoral responses in BALB/c mice following immunization with inactivated *P*. *berghei* XAT. BALB/c received three immunizations at two-week intervals using either glutaraldehyde-fixed *P*. *berghei* XAT-pRBCs or glutaraldehyde-fixed non-parasitized RBCs (npRBCs). Two weeks after the last immunization, all mice were challenged with *B*. *rodhaini*. **(A)** Survival curves and **(B)** Parasitemia were monitored until mice died. **(C)** Levels of *P*. *berghei* XAT-specific IgG, IgG1, and IgM antibodies were measured at day 7 pci. Data represent the mean ± SD from a single experiment (n = 5 mice per group). A two-tailed unpaired Student’s t-test was used for statistical comparisons of antibody levels between two groups. Statistical significance is denoted by asterisks (**p < 0.01, and ***p < 0.001).

To determine whether dead *P*. *berghei* XAT parasites could induce protective immunity against *B. rodhaini* infection, BALB/c mice were i.p. injected with inactivated *P. berghei* XAT-infected red blood cells (RBCs), followed by two booster injections at intervals of 14-days. Notably, after being challenged with *B. rodhaini*, all mice exhibited a rapid increase in parasitemia and succumbed to the infection within 8 days pci (**Figures 4A, B**). The immunized mice developed higher titers of specific antibodies against *P. berghei* XAT. In contrast, control mice that received injections of uninfected murine RBCs showed very low antibody levels (**Figure 4C**). These findings suggest that immunization with dead *P. berghei* XAT does not provide protective immunity against subsequent *B. rodhaini* infection.

### Effect of *P*. *berghei* XAT and *B*. *rodhaini* infection on splenic immune cells

To assess how co-infection influences splenocyte subsets associated with immune responses and disease pathogenesis, flow cytometric analysis was performed on day 7 pci. This analysis revealed alterations in the abundance of splenic B cells, T cells, macrophages, natural killer (NK) cells, and dendritic cells (DCs) (**Figure 1**). *Babesia* infection was associated with a reduction in the percentage of splenic T cells (**Figure 1A**) and B cells (**Figure 1B**) in Br and co-infected mice (T cells: Br vs Naïve, *p* = 0.017; PbXAT/Br14 vs Naïve, *p* = 0.017; B cells: Br vs Naïve, *p* = 0.036). DC populations were lowest in Br mice and significantly reduced compared to naïve mice (*p* < 0.001) and the PbXAT/Br14 group (*p* = 0.047, **Figure 1C**). Conversely, the percent population of NK cells showed a slight increase compared to Naïve mice, however, the increase was only significant in Br mice (*p* = 0.0010) compared to naïve controls (**Figure 1D**). Notably, macrophages in the spleens of co-infected mice exhibited a significantly higher percentage compared to mice with sole infection of *B. rodhaini* or other groups (**Figure 1E**). This increase was particularly pronounced in PbXAT/Br14 mice compared to Br mice (*p* < 0.001) and naïve mice (*p* = 0.009). CD4^+^ T cells were lower in Br and co-infected mice compared to Naïve controls. CD8^+^ cells did not show statistically significant differences among groups (*p* > 0.05, **Figures 1F, G**). Overall, the populations of adaptive immune cells (T and B cells) in co-infected mouse spleens were reduced, while those of innate immune cells (NK cells and macrophages) were increased compared to Naïve mice.

### Cytokine crosstalk and ROS/NO levels

**Figure 5:**
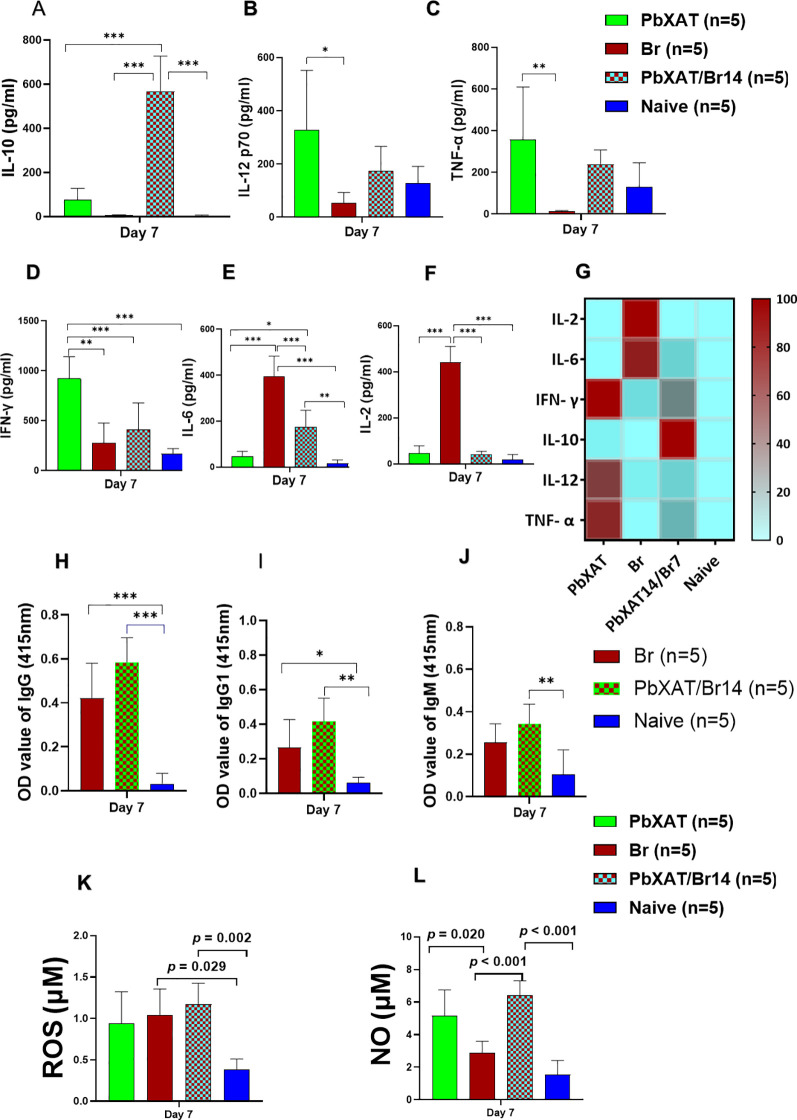
Serum cytokine antibody profiles in PbXAT, Br, PbXAT14/Br, and Naïve mice groups at day 7 pci. After primary infection with *P*. *berghei* XAT, mice were challenged with *B*. *rodhaini* at day 14. Serum collected on day 7 post-challenge was assayed for **(A)** IL-10, **(B)** IL-12p70, **(C)** TNF-α, **(D)** IFN-γ, **(E)** IL-6, and **(F)** IL-2. **(G)** A heatmap summarizes the secretion profile of all six cytokines. Measurement of serum **(H)** IgG, **(I)** IgG1, and **(J)** IgM levels, **(K)** ROS/RNS, and **(L)** NO levels at day 7 pci. Data represent the mean ± SD from a single experiment (n = 5 mice per group). Statistical significance was determined by ordinary one-way analysis of variance (ANOVA) followed by Tukey’s multiple comparison test. Asterisks denote statistical significance (*p < 0.05; **p < 0.01, and ***p < 0.001).

To evaluate systemic immune responses, serum antibody titers, cytokine profiles, and ROS/NO levels were quantified on day 7 post-infection (pi) in mice challenged with *B*. *rodhaini* following a primary *P*. *berghei* XAT infection. These data were subsequently compared with those from *B*. *rodhaini*, *P*. *berghei* XAT single-infected mice, as well as naïve mice. The interplay of cytokines in mice was assessed by measuring serum concentrations of IFN-γ, TNF-α, IL-6, IL-12p70, IL-2, and IL-10. Pro-inflammatory cytokines IL-6, IFN-γ, and TNF-α are major mediators associated with *Babesia* infection. (23, 46). However, a shift in cytokine balance was observed in co-infected mice, characterized by a decrease in IL-6, and IL-2 and an increase in IFN-γ, IL-12p70, TNF-α, and IL-10 levels compared to Br mice (**Figures 5A–F**). The observed shift in cytokines points towards the regulatory effect of primary *P*. *berghei* XAT infection. The increase in the level of IL-10 was significant in protected co-infected mice compared to Br mice, suggesting the protective role of IL-10 (**Figure 5A**) (46). IFN-γ was the highest in PbXAT mice, followed by co-infected, Br, and Naïve mice (**Figure 5D**). Br mice exhibited the highest levels of IL-6 and IL-2 among all mouse groups, and this increase was statistically significant (**Figures 5E, F**). Although co-infected mice exhibited slightly higher titers of IgG, IgG1, and IgM compared to the Br group, these differences were not statistically significant (**Figures 5H–J**). Overall, antibody levels remained low. At day 7, ROS levels were moderately elevated in the Br and PbXAT/Br14 groups compared to PbXAT and naïve mice, with the highest levels observed in the co-infected (PbXAT/Br14) mice. This increase in the PbXAT/Br14 group was not statistically significant. In contrast, naïve mice exhibited the lowest ROS levels (**Figure 5K**). Similarly, NO production was significantly (*p* = 0.007) increased in the co-infected group (PbXAT/Br14) compared to both single-infected (PbXAT and Br) and naïve groups, while naïve mice again showed the lowest levels (**Figure 5L**). Overall, co-infection resulted in enhanced NO and ROS levels compared to the Br group (**Figures 5K, L**).

### Co-infection mitigates *B*. *rodhaini*-induced splenic damage and splenomegaly

**Figure 6:**
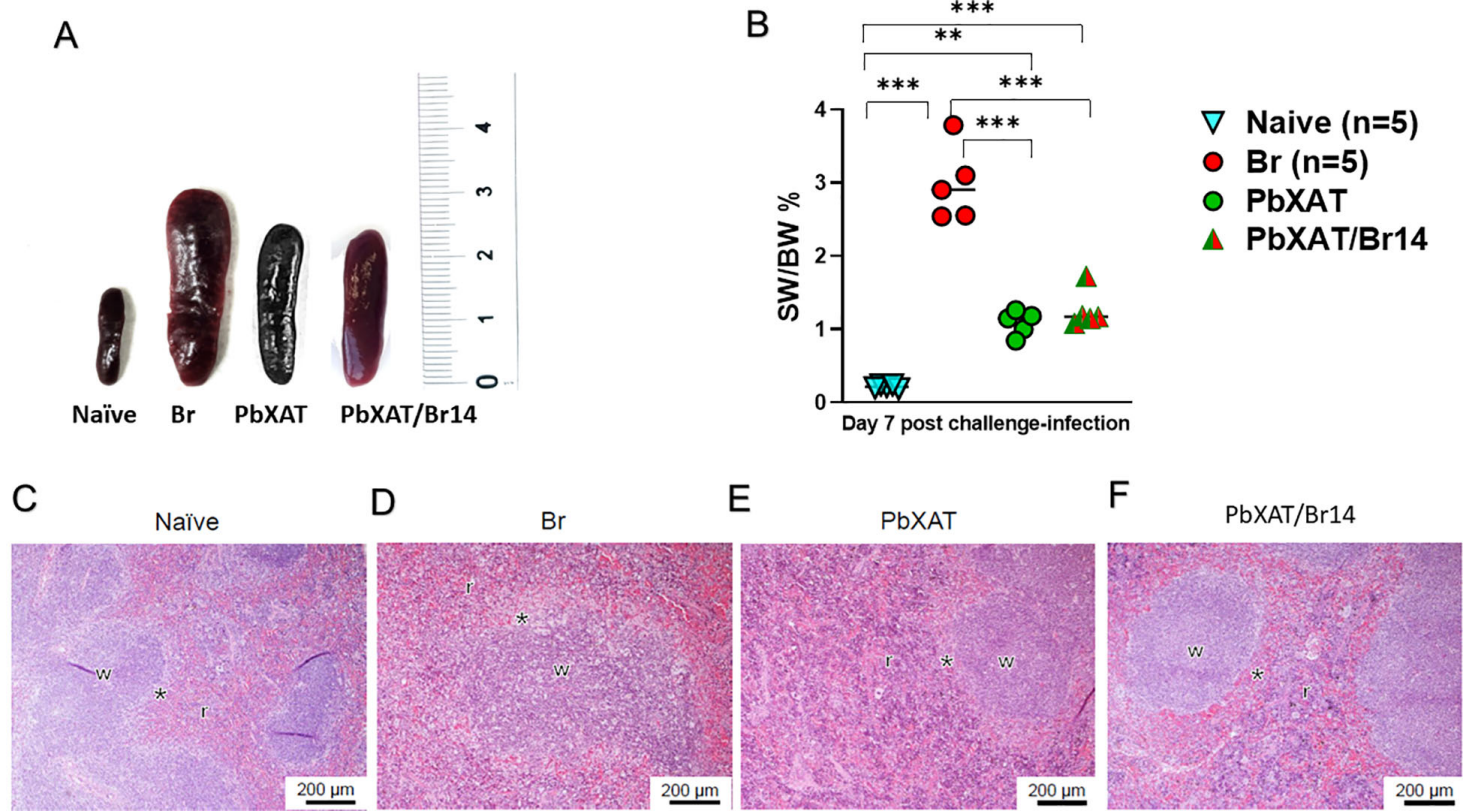
**(A)** Representative macroscopic images of spleens from Naïve, *B. rodhaini* (Br), PbXAT, and PbXAT/Br14 (co-infected) mice at day 7 post-challenge. **(B)** Splenic weight as a percentage of total body weight (SW/BW %) at day 7 post-challenge. Br mice exhibited extreme splenomegaly compared to Naïve mice (*p* < 0.001), whereas co-infection significantly reduced this pathological enlargement (*p* < 0.001 vs. Br). Sections collected on day 7 post-challenge infection were stained with H & E, 200×. **(C)** Naïve spleen section showed distinct red (r) and white (w) pulps. **(D)** The Br mouse spleen sample indicated a blurred boundary between red and white pulp because of repletion of immune cells in the red pulp. **(E, F)** In PbXAT and PbXAT/Br14 mouse spleen, the marginal zone (*) is clearly distinguished by the white pulp. Asterisks indicate statistical significance asterisks (**p < 0.01, and ***p < 0.001).

Gross examination revealed that spleens from *B*. *rodhaini*-infected mice exhibited profound splenomegaly by day 7 pci compared with naïve controls (**Figure 6A**). This observation was confirmed by a significant increase in the SW/BW ratio, which reached approximately 3% in the Br group compared to the negligible ratios in naïve mice (*p* < 0.001; **Figure 6B**). In contrast, PbXAT and PbXAT/Br14 groups appeared smaller, comparable, and showed significantly reduced SW/BW ratios compared with *B*. *rodhaini*-infected mice, indicating significant attenuation of splenomegaly compared to the Br group (*p* < 0.001).

H&E-stained spleen sections from naïve mice displayed normal architecture, with clearly demarcated red and white pulp regions (**Figure 6C**). In contrast, spleens from *B*. *rodhaini*-infected mice exhibited disruption of this organization, with indistinct red and white pulp boundaries accompanied by marked cellular accumulation within the red pulp (**Figure 6D**). A mild disruption of the marginal zone was seen in PbXAT spleen sections (**Figure 6E**). Notably, co-infected mice retained more defined splenic architecture, with an intact marginal zone clearly separating the white pulp (**Figure 6F**). The most profound alteration in splenic structure and marginal zone integrity was observed in the *B*. *rodhaini* group, characterized by a substantial depletion of marginal zone cellularity. Overall, these findings suggest that co-infection decreased the splenic damage.

### Cross-protection against *B*. *rodhaini* conferred by *P*. *berghei* XAT is independent of B and T lymphocytes

**Figure 7:**
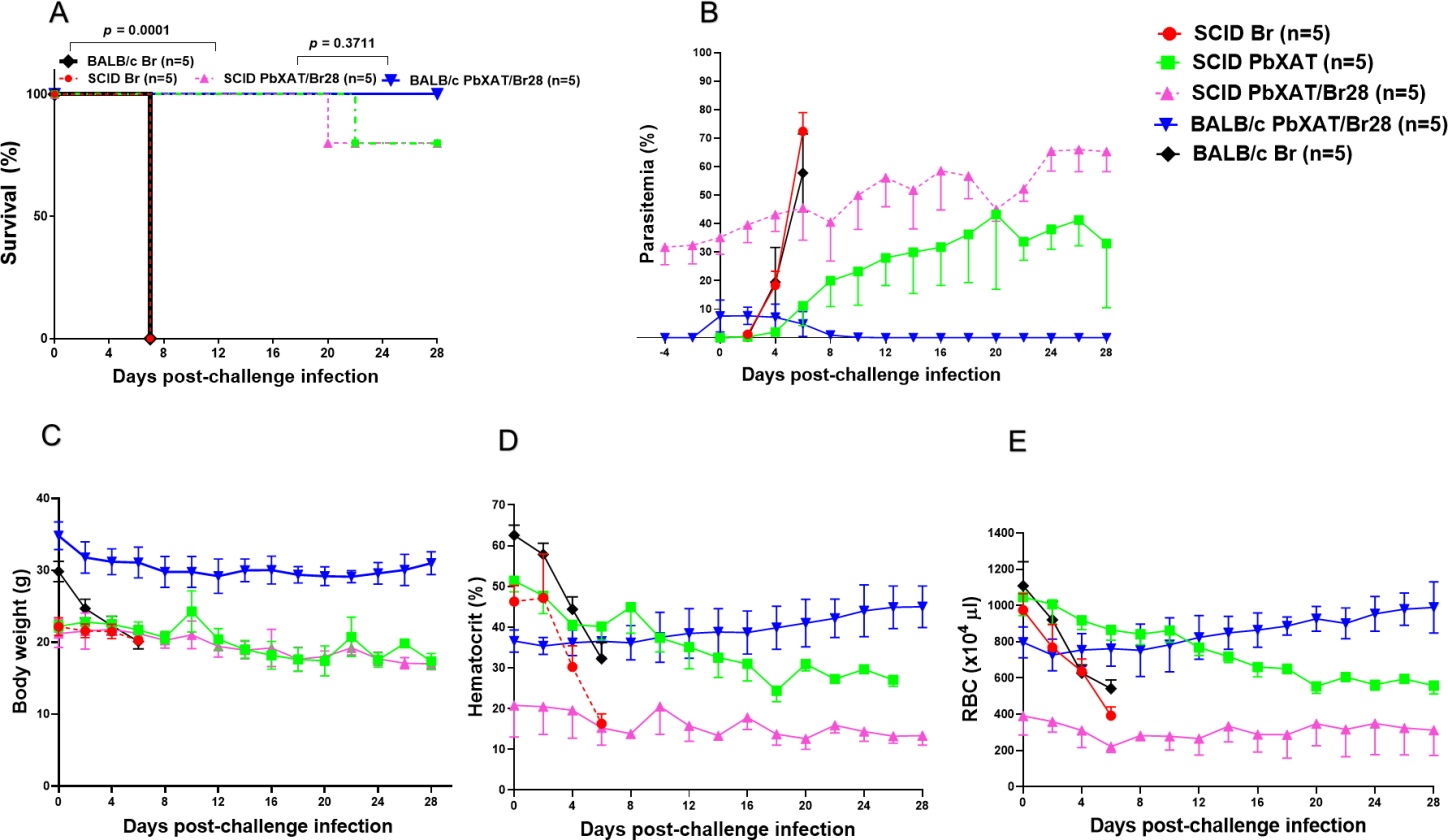
Disease progression in all SCID or BALB/c mice from all experimental groups. Mice were first infected with *P. berghei* XAT and subsequently challenged with *B. rodhaini* at 28 post-primary infection. **(A)** survival curve, **(B)** parasitemia, **(C)** body weight, **(D)** hematocrit, **(E)** red blood cell (RBC), values were monitored until death in Br, and all other groups (PbXAT/Br28, PbXAT, and Naïve) were observed for 28 days. Data represent mean ± standard deviation (SD) from five mice per group (n = 5), and are representative of two independent experiments. Survival data were analyzed with the Kaplan-Meier non-parametric model. Group comparisons were performed using one-way ANOVA with Tukey’s *post hoc* test or a two-tailed Student’s t-test.

To examine the contribution of B and T lymphocytes to protection against lethal *B*. *rodhaini* infection, SCID mice were first infected with *P*. *berghei* XAT and subsequently challenged with *B*. *rodhaini* 28 days later. Disease progression following challenge was compared between SCID mice chronically infected with *P*. *berghei* XAT and non-immunized SCID control mice. Notably, the parasitemia kinetics observed in SCID mice differed significantly from those seen in immunocompetent BALB/c mice (**Figure 7**). Following *B*. *rodhaini* infection, control SCID mice showed a rapid rise in parasitemia, exceeding 80%, and all mice died within one week of challenge (**Figures 7A, B**). In contrast, SCID mice chronically infected with *P*. *berghei* XAT survived the *B*. *rodhaini* challenge for more than four weeks (**Figures 7A, B**). Although they persistently maintained high parasitemia of more than 60% (**Figure 7B**). Not only the co-infected mice but also PbXAT SCID mice showed persistent parasitemia in high levels (30-40%) for at least 28 days (**Figure 7B**). Protection conferred by *P. berghei* XAT was lower in SCID mice (80% survival rate) in comparison to BALB/c mice (100% survival rate), but the reduction in survival was statistically insignificant (*p* = 0.3711). The lower survival rates of SCID mice compared to BALB/c mice demonstrate the role of lymphocytes and the adaptive immune system in combating *B*. *rodhaini* infection (**Figure 7A**). Importantly, the majority of SCID mice chronically infected with *P. berghei* XAT survived the challenge with *B. rodhaini*, whereas all non-immunized SCID mice quickly succumbed to the *B*. *rodhaini* infection (*p* = 0.0001). Although co-infected SCID mice experienced reductions in body weight, hematocrit, and RBC counts, these declines were gradual. In contrast, mice infected with *B*. *rodhaini* alone exhibited a sharp, rapid decline in measured parameters (**Figures 7C–E**). These results indicate that the protective state induced by *P. berghei* XAT infection against the lethality of *B. rodhaini*-challenge is not impaired in the absence of B and T lymphocytes, suggesting a crucial role for the innate immune system in mediating cross-protection.

### Macrophage, but not NK cell depletion, impairs *P*. *berghei* XAT-mediated protection against *B*. *rodhaini*

To understand the role of the innate immune system in *P. berghei* XAT-mediated cross-protection against *B. rodhaini*, we next investigated whether depleting innate immune cells, specifically NK cells and macrophages/monocytes, would diminish this protection. In two independent experiments, NK cells or macrophages/monocytes were depleted from BALB/c mice through intraperitoneal administration of anti-asialo GM1 antibody or clodronate-encapsulated liposomes, respectively. Depletion efficacy was subsequently confirmed by flow cytometry (**Figures 2A, B**). Depletion of NK cells resulted in a moderately higher peak parasitemia (10%) compared to control antibody-treated mice, which subsequently declined by the second week following challenge infection. Consistently, survival did not differ significantly between NK cell-depleted and control antibody-treated (*p* > 0.05) (**Figures 2C, D**). In contrast, mice treated with clodronate liposomes exhibited significantly higher parasitemia (>30%) than those receiving PBS liposomes. As a result, over 60% of these mice succumbed to the challenge infection within two weeks (*p* = 0.0085; **Figures 2F, G**). Mice depleted of NK cells or macrophages exhibited a marked but transient reduction in body weight during the first two weeks post-challenge, while control mice maintained stable body weight throughout the infection period (**Figures 2E, H**). Our results showed that while depletion of NK cells did not significantly abrogate protection, selective macrophage depletion markedly abolished cross-protection, rendering mice vulnerable to lethal *B*. *rodhaini* challenge-infection. This finding demonstrates a critical role for macrophages in providing protective immunity against *B*. *rodhaini*. This is consistent with previous studies identifying macrophages, rather than NK cells, as the primary mediators of cross-protection (22, 25).

### Depletion of macrophages impairs the balanced cytokine, NO and ROS secretion required for cross-protection

**Figure 8:**
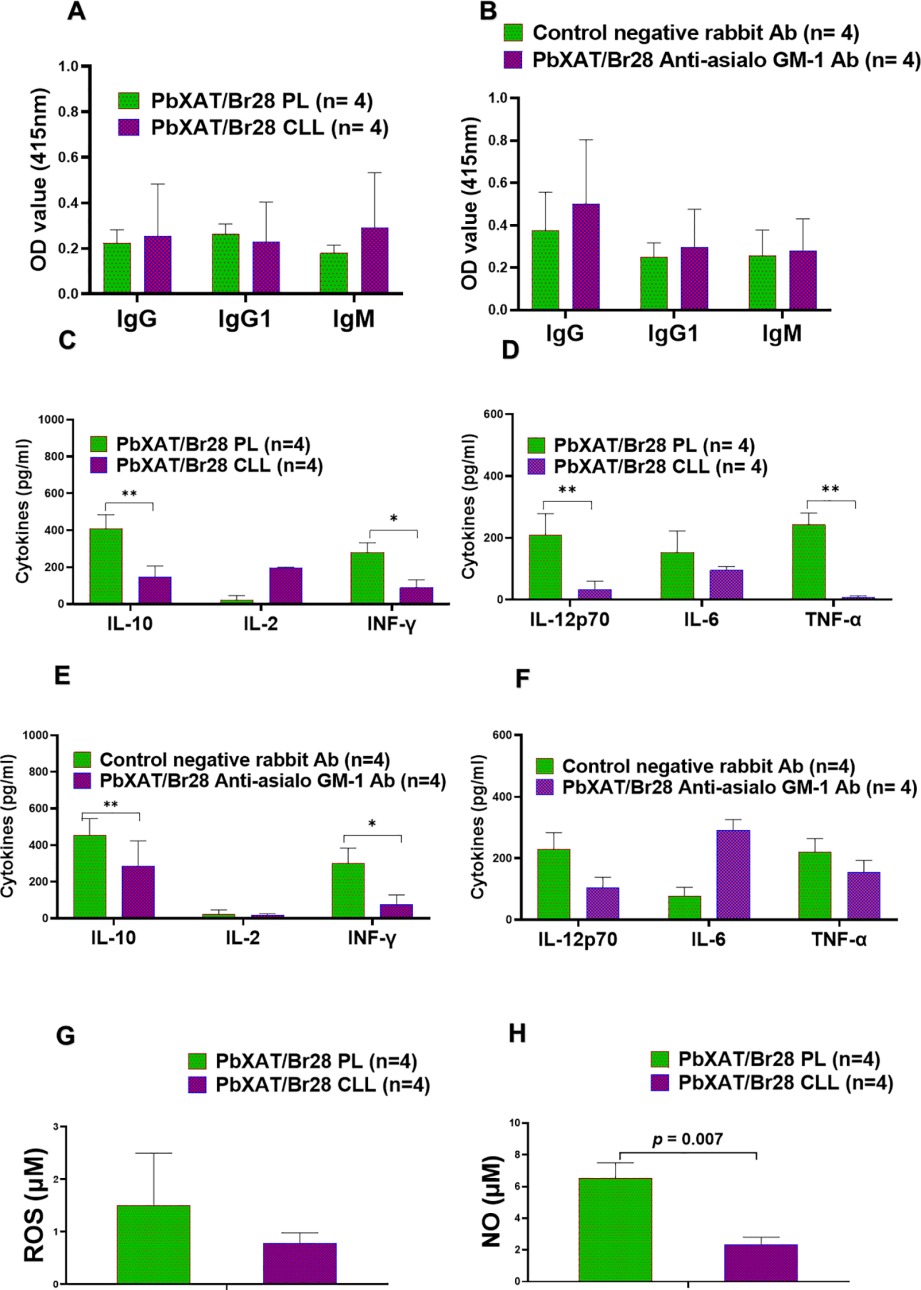
Antibody and cytokine responses measured at day 7 pci following macrophage or NK cell depletion in mice challenged with *B. rodhaini* 28 days after primary infection with *P. berghei* XAT infection. **(A)** Serum levels of *P. berghei*XAT-specific IgG, IgG1, and IgM in PbXAT/Br28 mice treated with PBS liposomes (PL) or clodronate liposomes (CLL). **(B)** *P. berghei* XAT-specific IgG, IgG1, and IgM levels in PbXAT/Br28 mice treated with control rabbit antibody or anti-asialo GM-1 antibody. **(C, D)** Serum cytokine concentrations in PbXAT/Br28 mice following macrophage depletion, including **(C)** IL-10, IL-2, and IFN-γ, and **(D)** IL-12p70, IL-6, and TNF-α. **(E, F)** Serum cytokine levels in PbXAT28/Br mice following NK cell depletion, including **(E)** IL-10, IL-2, and IFN-γ, and **(F)** IL-12p70, IL-6, and TNF-α. **(G)** ROS and **(H)** NO levels in mice after macrophage depletion. Data represent the mean ± SD from a single experiment (n = 5 mice per group). Group comparisons were performed using one-way ANOVA with Tukey’s *post hoc* test or a two-tailed Student’s t-test. Asterisks indicate statistical significance asterisks (*p < 0.05, and **p < 0.01).

To determine whether the absence of macrophages/monocytes compromises the humoral and cytokine responses to infection, serum antibody levels and cytokine production were assessed at day 6 following *B*. *rodhaini* challenge. In parallel, levels of nitric oxide (NO) and reactive oxygen species (ROS) were also measured. Despite the marked differences in parasitemia profiles, we observed no significant variations in the antibody responses of mice treated with clodronate liposomes compared to those receiving PBS-liposomes (**Figure 8A**). A similar lack of significant difference was observed when comparing anti-asialo GM1-treated mice to their controls (**Figure 8B**). Specifically, IgG, IgG1, and IgM levels between the PbXAT/Br28 CLL and PbXAT/Br28 PL groups were non-significant (*p* = 0.248, 0.934, 0.248, respectively). Similarly, NK cell depletion via anti-asialo GM1 administration did not significantly alter antibody titers when compared to the rabbit IgG control group. These results suggest that the loss of these innate effector cells impairs parasite control without compromising the initial humoral response.

Following macrophage depletion using clodronate liposomes, we observed significantly lower production of IFN-γ, TNF-α, IL-10, IL-12p70, and IL-6 compared to PBS-liposome-treated controls (**Figures 8C, D**). Specifically, IL-10 (*p* = 0.006), IFN-γ (*p* = 0.02), IL-12p70 (*p* = 0.007), and TNF-α (*p* = 0.003) levels were significantly reduced in CLL-treated mice. In contrast, PBS-liposome-treated control mice maintained higher levels of these cytokines. This dysregulated cytokine response may contribute to increased lethality from *B*. *rodhaini* (**Figures 8C, D**, **2F, G**). Moreover, a decrease in serum ROS and NO levels was observed following macrophage depletion in co-infected mice (**Figures 8G, H**). While the reduction in ROS was not statistically significant, NO levels were significantly reduced (*p* = 0.007), consistent with previous findings (47). Together, these findings indicate that depletion of macrophages/monocytes disrupts the balanced cytokine milieu, reduces ROS and NO production, exacerbates parasitemia, and ultimately results in fatal outcomes.

Preliminary NK cell depletion experiments revealed that while anti-asialo GM1-treated mice exhibited elevated parasitemia and mortality compared to controls, cross-protective immunity was not completely abolished. Yoshida et al. (48) reported significantly lower levels of IFN-γ, TNF-α, and IL-6 from liver and spleen cells following NK depletion. Similarly, we found that secretion of IFN-γ, TNF-α, and IL-10 was notably reduced in anti-asialo GM1-treated mice compared to controls (**Figures 8E, F**). In particular, IL-10 (*p* = 0.008) and IFN-γ (*p* = 0.03) levels were significantly decreased following NK cell depletion. Interestingly, while TNF-α and IL-12p70 levels also showed a decreasing trend, NK cell depletion resulted in an apparent compensatory increase in IL-6 levels compared to controls (**Figure 8F**). The significant reduction in IFN-γ underscores the role of NK cells in the early cytokine crosstalk required for optimal defense.

## Discussion

The incidence of co-infections is increasing, driven by expanding vector populations associated with climate change and intensified human disruption of natural ecosystems (12). Among the most clinically significant vector-borne pathogens, the intraerythrocytic protozoa, specifically *Plasmodium* and *Babesia* spp. (49), provided the impetus for the present study. Here, we seek to elucidate the immunological interplay and host-pathogen interactions that emerge during co-infections. To investigate these dynamics, we used the non-lethal *P. berghei* XAT strain, derived from *P*. *berghei* NK65, which has proven valuable for studying malaria immunopathology (50). The choice of the attenuated primary infection likely allowed sufficient time for the host to establish a balanced innate immune response. In contrast, lethal strains such as *P. berghei* ANKA, which model *P. falciparum* infection, induce hyper-inflammatory responses characterized by excessive production of IFN-γ and TNF-α (50). Such virulent models often induce experimental cerebral malaria (ECM) or lethal systemic inflammation. This may overwhelm counter-regulatory mechanisms such as IL-10 production before cross-protection can be established (51, 52). Malaria severity often reflects the timing and resolution of host immune responses rather than parasite load alone (53). Thus, the window for heterologous immunity may depend on a primary infection that is sufficiently inflammatory to prime macrophages, yet controlled enough to avoid early immunopathology and host mortality. In addition, the protection acquired from the primary infection will also depend on the timing of the secondary infection. For babesiosis, we utilized *Babesia rodhaini* (Australian strain), a rodent parasite closely related to the primary human pathogen *Babesia microti*, and capable of infecting human erythrocytes *in vitro* (54). This strain provides a critical model, as infections with *B*. *microti* are typically self-limiting, whereas *B*. *rodhaini* causes a virulent and often fatal disease in mice, allowing for the study of severe babesiosis pathophysiology (23, 55), providing a stringent framework to evaluate the protective efficacy of heterologous immunity.

To our knowledge, this is the first report to explore whether an ongoing *P. berghei* XAT infection alters the course of lethal *Babesia* in a co-infection mouse model. This finding expands upon the reciprocal relationship suggested by previous work, which demonstrated that *Babesia* infection protects against *Plasmodium* in both macaques and murine models (21, 22, 29). The *P*. *berghei* XAT strain induces a self-resolving infection, with parasitemia typically reaching up to 10% before complete recovery within approximately four weeks (50, 56, 57). Consistent with these reports, our data showed comparable parasitemia kinetics (**Figure 3B**), with peak parasitemia in *P*. *berghei* XAT-infected mice reaching ~14%. Crucially, this primary infection significantly attenuated the high parasitemia and severe anemia typically induced by *B*. *rodhaini* challenge, a pattern of attenuation previously noted in other co-infection studies (22, 25, 58). In contrast, Br mice exhibited rapid parasite proliferation and exacerbated anemia (**Figures 3B–D**), consistent with previous reports of fulminant babesiosis (59). This protective effect was dependent on a live primary infection, as inoculation with dead *P*. *berghei* XAT parasites failed to confer protection, similar to previous findings where dead *Babesia* parasites did not protect against lethal challenge (22, 25). This aligns with the established principle that live parasites elicit a more potent and protective immune response than non-viable counterparts, potentially due to sustained antigenic exposure to trigger a sufficiently potent immune response (60, 61). To investigate whether simple resource competition between parasites explained the attenuation in *Babesia* parasitemia, as inter-pathogen resource competition is possible (12). Our qPCR results confirmed that both peripheral blood and spleen samples from co-infected mice were positive for *B*. *rodhaini*, demonstrating that the observed parasitemia attenuation in co-infected mice represents an immune-mediated reduction in total parasite burden rather than a failure of infection establishment (**Figures 3F–I**). Moreover, De Roode et al. (62) demonstrated that parasite virulence is positively correlated with competitive fitness in co-infections, with more virulent strains generally outcompeting less virulent ones. In our model, *P*. *berghei* XAT is less virulent than *B*. *rodhaini*, further supporting the conclusion that the protective mechanism is driven by the host immune response.

The spleen plays a central role in regulating inflammatory and anti-parasitic responses and in clearing infected erythrocytes. Thus, its structural integrity is a vital indicator of disease severity (63–65). Our histopathological analysis revealed that the protected mice, which had lower *B*. *rodhaini* burden, exhibited significantly preserved splenic architecture (**Figures 3G**, **6F**). In contrast, the extensive tissue disruption and severe histopathological damage in lethal *B*. *rodhaini* single-infected mice, which had higher parasitic loads (**Figures 3G**, **6D**; 66). The observed splenic alterations and characteristic splenomegaly are not merely indicators of damage. They also reflect intense innate immune activation, marked by the expansion and recruitment of monocytic and phagocytic populations critical for clearing parasitized erythrocytes (67, 68). Such structural reorganization facilitates the mechanical and immunological elimination of *Babesia*-infected red blood cells, thereby influencing disease outcome (47, 69). Splenic analysis revealed that co-infected mice had significantly larger spleens than naïve mice, reinforcing the beneficial nature of this response (**Figures 6A, B**). Furthermore, flow cytometry demonstrated a significant expansion of macrophages in co-infected mice compared to Br mice (**Figure 1E**). The noteworthy optical density (OD) observed at Day 7 is consistent with the rapid induction of anti-erythrocyte antibodies reported in *B*. *rodhaini* models (70), and the polyclonal B-cell activation characteristic of acute apicomplexan infections (71, 72). This early increase may also reflect a primed humoral response following prior *P*. *berghei* XAT infection (73). Consistent with previous reports, prior exposure can induce heterologous immunity; however, cross-reactive antibodies alone do not fully account for the observed protection (20). Indeed, high antibody titers do not necessarily confer protection against a lethal *Babesia* challenge (74). Given that antibody levels in protected co-infected mice did not significantly exceed those in the lethal single-infection group, our data support an antibody-independent mechanism for cross-protection (**Figures 5H–J**). The substantial splenic remodeling may also compromise germinal center integrity, impairing B cell maturation and potentially affecting the quality of antibody and adaptive immune responses during concurrent malaria infection (75). One possible explanation is the development of *Babesia*-induced splenic pathology, which impairs lymphocyte function and limits antibody production, as previously reported in murine *B*. *microti*-*Borrelia burgdorferi* co-infection models (36, 76). Our immunophenotyping data confirm these findings, revealing a decline in B and T lymphocyte (**Figures 1A, B**), a pattern consistent with other acute *Babesia* co-infection studies (Fig. 36, 69). This shift in cellular composition, specifically the significant increase in the splenic macrophage population alongside a reduction in lymphocytes, highlights a pivotal reliance on innate effector mechanisms. Primarily mediated by macrophage-driven responses rather than classical adaptive immunity during the acute phase of infection. In the broader context of immunological memory, cross-protection typically arises through two distinct pathways: antigen-specific cross-reactivity mediated by T cells and antibodies (77, 78), or a non-specific, heightened state of innate readiness orchestrated by activated macrophages during a primary stimulus (79). Our data strongly suggest that the latter mechanism operates in the *P*. *berghei* XAT-mediated defense against *B*. *rodhaini*.

To precisely delineate the contribution of immune cells to the observed cross-protection, we further dissected the innate compartment using SCID models and selective depletion of NK cells and macrophages. This approach helped to directly interrogate the cellular mediators underlying heterologous immunity. We found that SCID mice, which lack functional B and T cells, retained significant protection against *B*. *rodhaini* following *P*. *berghei* XAT infection (**Figure 7A**), ruling out the essential role for adaptive immunity. This finding is in agreement with previous reports indicating that the absence of B and T lymphocytes does not significantly compromise resistance to *Babesia* WA1 or *Plasmodium* spp., and the early protective responses in both babesiosis and malaria are largely antibody independent (80–83). However, the recrudescence of parasitemia observed in B-cell-deficient mice approximately 30 days post-infection showed that the antibodies are critical for the final clearance of residual parasitemia and preventing recurrence (84). Skariah et al. (26) also showed that B cells are important for the clearance of parasites. Schreiber et al. (85) used SCID mice to demonstrate that macrophage activation can occur independently of T cells, describing an important mechanism for innate resistance to infection. While the innate immune system is sufficient to confer cross-protection against lethal challenge, the adaptive immune response remains important for optimal parasite control and long-term resolution. During the establishment phase of infection, antibodies contribute to host defense by binding free sporozoites, thereby preventing erythrocyte invasion (83, 86). Additionally, during the resolution phase, antibodies promote phagocytosis of merozoites and infected red blood cells through a mechanism known as antibody-dependent cellular inhibition (ADCI). Thereby, facilitating the clearance by phagocytic cells (87). Consistent with these roles, our results show that SCID mice, which lack functional B and T cells, exhibited lower survival rates and persistently higher parasitemia compared to immunocompetent BALB/c mice (**Figures 7A, B**). This demonstrates the contribution of adaptive immunity in resolving parasitemia and achieving complete parasite clearance.

Macrophages are central mediators of the host immune response against hemoparasitic infections, including malaria and babesiosis. They function not only through the phagocytosis of infected red blood cells (iRBCs) but also by shaping the local immune environment via the production of both pro- and anti-inflammatory cytokines (88). The phagocytic function of macrophages is a critical protective mechanism; they internalize *Plasmodium* and *Babesia* iRBCs via both opsonic pathways, involving antibodies and complement, and non-opsonic pathways mediated by pattern recognition receptors (PRRs) and adhesion molecules like ICAM-1 and CD36 (89). As “sensors of danger,” macrophages utilize Toll-like receptors (TLRs) and the MyD88 adaptor molecule to detect endogenous signals from necrotic debris (90–93). Macrophages express pattern recognition receptors (PRRs) on their surface, which allow them to identify and engulf pathogens. Among these, Toll-like receptors (TLRs) represent an important class of PRRs essential for initiating innate immune responses (89). Importantly, this sensing and subsequent activation can occur in the absence of lymphocytes (92), further validating our SCID mouse data, which demonstrate that these processes do not critically depend on the adaptive immune response. This property likely contributes to the rapid initiation of protective responses early during infection. The cytokine milieu further shapes the macrophage activation. Cytokines produced by immune cells can drive macrophage differentiation into distinct functional phenotypes (94, 95). Classically activated macrophages (M1) arise in response to IFN-γ and TNF-α and exhibit potent microbicidal activity. Originally described by Mackaness (96), these macrophages secrete high levels of pro-inflammatory mediators. In our co-infection model, higher levels of TNF-α and IFN-γ (**Figures 5C, D**) likely promote classical activation, resulting in effector macrophages with an enhanced capacity to clear *Babesia*-pRBCs (96, 97). The protective role of macrophages in babesiosis is well-documented, with soluble factors often mediating parasiticidal activity (83, 98, 99). Our findings suggest that *P*. *berghei* XAT infection serves as a potent primer for these innate responses, enhancing the host’s ability to mount an accelerated inflammatory defense. Franklin et al. (100) showed that TLR9-MyD88 signaling is essential for the production of IL-12 and IFN-γ and for enhancing cellular responsiveness to TLR stimulation. In agreement with these reports, our cytokine analysis revealed significantly elevated IL-12p70 and TNF-α levels in *P*. *berghei* XAT-infected and co-infected mice (**Figures 5B, C**). This supports the notion that prior exposure to *Plasmodium* antigens sensitizes innate immune cells, particularly macrophages, to mount an early inflammatory response to a secondary *Babesia* challenge.

Natural killer (NK) cells are important early producers of IFN-γ during parasitic infections and play a crucial role in initiating immune responses (101). The protective role of macrophages appears to rely on a positive feedback loop between macrophages and NK cells, similar to the T-cell-independent pathways described in *Toxoplasma gondii* infections. Upon parasite recognition, macrophage-derived IL-12 stimulates NK cells to produce IFN-γ, which in turn activates macrophages and enhances their production of pro-inflammatory cytokines (TNF-α), reactive oxygen species, and nitrogen radicals, including nitric oxide (NO), thereby increasing their microbicidal capacity (102–108). Previous findings by Aguilar-Delfin et al. (109) similarly demonstrated that protection against pathogenic *Babesia* WA1 depends largely on macrophage-NK cell interactions mediated by early IL-12 and IFN-γ responses and the induction of macrophage-derived effector molecules. In our study, NK cell depletion resulted in significantly reduced levels of IFN-γ, TNF-α, and IL-10 (**Figures 8E, F**), accompanied by increased parasitemia and mortality (**Figures 2C, D**), indicating impaired activation of downstream effector cells such as macrophages (110, 111). Disruption of this macrophage-NK cell crosstalk, either through NK or macrophage depletion, led to an imbalanced cytokine response and compromised parasite control, ultimately promoting disease progression (112). Collectively, our data suggest that *P*. *berghei* XAT infection may prime innate immune responses, enhancing macrophage activation and contributing to improved cross-protective immunity.

Pro-inflammatory cytokines play a crucial role in controlling parasite replication. Early production of IFN-γ is essential for protection against experimental cerebral malaria (ECM), whereas delayed or insufficient IFN-γ responses are associated with increased mortality (101, 113). IL-12 further supports this response by promoting IFN-γ production and enhancing innate immune activation. However, excessive production of pro-inflammatory cytokines such as IFN-γ, TNF-α, and IL-12 can lead to severe immunopathology. Elevated levels of these mediators are associated with tissue damage, partly through the upregulation of endothelial adhesion molecules such as ICAM-1, VCAM-1, and P-selectin, which promote sequestration of infected erythrocytes and leukocytes (114, 115). Such dysregulated inflammation contributes to complications, including cerebral malaria and severe anemia. In our study, co-infected mice exhibited elevated levels of IL-12p70, IFN-γ, and TNF-α, suggesting enhanced activation of innate immune responses (**Figures 5B–D**). This cytokine profile is consistent with improved parasite control, likely mediated by macrophage activation and increased microbicidal activity. Conversely, IL-6 and IL-2 levels were highest in *B*. *rodhaini*-infected mice (**Figures 5E, F**), cytokines often associated with exacerbating the disease in lethal malaria and babesiosis (29, 116–118).

A balanced immune response, characterized by controlled cytokine production, facilitates pathogen clearance while minimizing tissue damage. In contrast, dysregulated release by innate or adaptive immune cells can result in a harmful cytokine storm (119, 120). Infection-driven hyperactivation of immune cells promotes the release of pro-inflammatory mediators, which contribute to tissue damage (121). Therefore, disease severity is often a manifestation of disproportionate inflammatory responses, which can persist even after pathogen clearance (122). To mitigate collateral damage from inflammation, counter-regulatory mechanisms are essential. The anti-inflammatory cytokine IL-10 serves this function by maintaining a balance between effective Th1-mediated antiparasitic immunity and immunopathology (123). However, IL-10 acts as a double-edged sword in malaria-associated pathology, particularly in the development of severe anemia. Low levels of IL-10 can suppress erythropoiesis either directly by inhibiting erythroid progenitor cell proliferation or indirectly by reducing erythropoietin production (124–126). Conversely, adequate IL-10 production can protect against malarial anemia by restraining excessive pro-inflammatory cytokines such as TNF-α, thereby preserving bone marrow erythropoietic function and limiting the destruction of uninfected red blood cells (127, 128). Thus, while IL-10 is critical for limiting hyperinflammation and tissue injury, its dysregulated or prolonged production may contribute to the pathogenesis of severe anemia (123). In our study, co-infected mice exhibited elevated levels of IL-12p70, IFN-γ, and TNF-α compared to *B*. *rodhaini*-infected mice, indicating enhanced activation of pro-inflammatory pathways (**Figures 5B–D**) (22, 29). Crucially, protected co-infected mice also showed significantly higher IL-10 levels, which likely served to counterbalance this potent pro-inflammatory burst and limit immunopathology (**Figure 5A**) (95, 129). This coordinated cytokine profile likely contributes to effective parasite control while preventing excessive tissue damage. In the context of our findings, despite the potential for IL-10 to contribute to anemia, co-infected BALB/c mice exhibited preserved hematocrit and RBC counts compared to the lethal single-infection group (**Figures 3D, E**; **Figure 5A**). This suggests that the protective benefits of IL-10 in controlling inflammation outweighed its potential suppressive effects on erythropoiesis. Consistent with this regulatory framework, macrophage functional polarization is largely dictated by the balance between IL-12 and IL-10 production. Ultimately, influencing whether a macrophage shifts toward a microbicidal (M1) or a regulatory (M2) state is largely dictated by this balance between IL-12 and IL-10 production (130). This cytokine balance in our protected co-infected mice suggests a protective homeostasis that prevented the splenic pathology observed in single-infected Br mice (**Figures 5A–D**, **6A–F**).

In addition to their role in cytokine regulation, macrophages contribute directly to parasite clearance through the production of reactive oxygen and nitrogen species (ROS/RNS), particularly nitric oxide (NO). Upon activation, the synergistic action of TNF-α and IFN-γ enhances NO generation, a primary effector mechanism for intracellular parasite killing (131). These reactive intermediates, together with other macrophage-derived soluble mediators, promote the degeneration of intraerythrocytic *Plasmodium* and *Babesia*. Thereby, limiting replication within the red blood cells (132, 133). Following phagocytic uptake of infected erythrocytes, these parasites are exposed to intracellular killing mechanisms, including oxidative burst and NO-mediated cytotoxicity, which collectively contribute to effective parasite clearance (89). In our study, co-infected mice exhibited elevated TNF-α levels, suggesting that enhanced macrophage activation and NO production contributed to parasite control (**Figures 5C, D**, **3B**). This likely reflects a synergistic interaction between TNF-α and IFN-γ that promotes efficient clearance of parasitized red blood cells while remaining sufficiently regulated to avoid excessive immunopathology. However, while ROS and NO are essential for parasite killing, their overproduction can exacerbate malarial pathophysiology (134). Thus, optimal host protection depends on a balanced regulation of macrophage effector functions. Our results demonstrate a significant increase in these microbicidal mediators in co-infected mice, which is abolished upon macrophage depletion (**Figures 5K, L**, **8G, H**). This provides indirect evidence that the expanded macrophages are functionally active and are the source of the oxidative burst required for parasite control.

**Figure 9:**
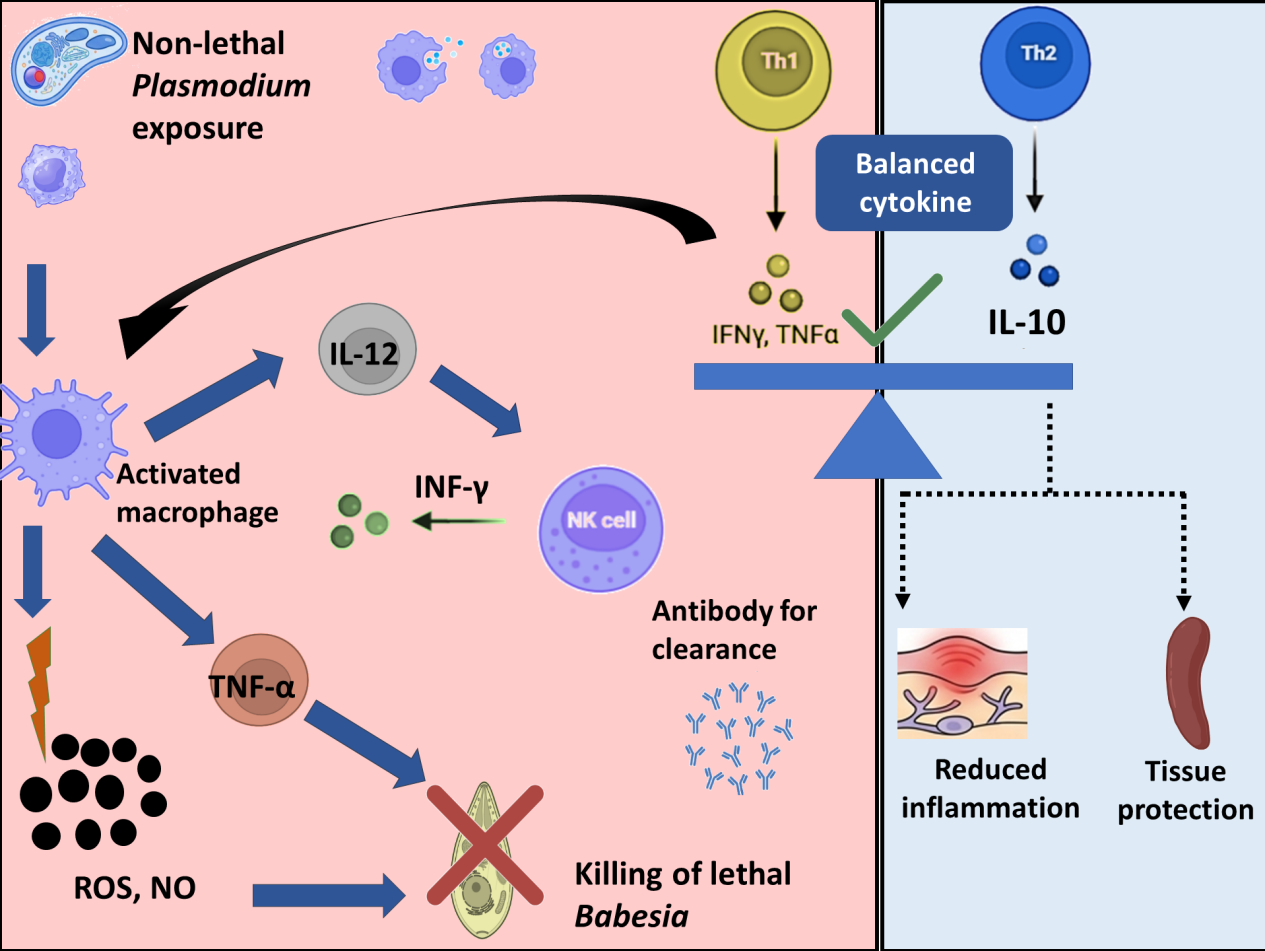
Schematic model of the proposed immune mechanism underlying cross-protection. Non-lethal *Plasmodium* exposure activates macrophages, leading to the production of IL-12, which stimulates natural killer (NK) cells to secrete IFN-γ. IFN-γ, together with Th1-derived cytokines, further enhances macrophage activation, promoting the release of TNF-α and the generation of reactive oxygen and nitrogen species (ROS/NO), ultimately contributing to the killing of *B. rodhaini*. Simultaneously, a Th2-associated response induces IL-10 production, which counterbalances excessive inflammation by regulating pro-inflammatory cytokine activity. This coordinated interplay between pro-inflammatory (IL-12, IFN-γ, TNF-α) and anti-inflammatory (IL-10) pathways establishes a balanced cytokine environment. This enables effective control of *B*. *rodhaini* infection with reduced immunopathology and improved host protection.

The critical role of macrophages in maintaining cytokine balance was evident in macrophage-depleted mice. Consistent with the previous report (47), depletion of macrophages resulted in significantly reduced levels of key cytokines, including IFN-γ, TNF-α, IL-10, IL-12p70, and IL-6. This impaired cytokine response was associated with increased parasitemia and mortality, indicating a failure to mount an effective and regulated immune response. Macrophages are also a major source of IL-10, and NO/ROS production (47). Their absence disrupts anti-inflammatory regulation, leading to uncontrolled disease progression (88, 123, 135). Research indicates that depletion of macrophages during infection markedly impairs the control of *Plasmodium* and *Babesia*, exacerbating clinical symptoms and leading to early death, as observed in our study (**Figure 2C**) and consistent with previous reports (135–137). In addition to cytokine dysregulation, macrophage depletion also resulted in increased parasitemia and decreased ROS/NO production (**Figures 2D**, **7C–H**). These findings clearly demonstrate that these cells are essential for removing parasitized erythrocytes, likely through both opsonic and non-opsonic phagocytosis pathways (138, 139). They also produce microbicidal mediators that contribute to parasite killing (83, 98). Collectively, these data indicate that macrophage depletion severely compromises host resistance to *B*. *rodhaini*, highlighting the critical role of macrophage-mediated phagocytosis and effector functions in limiting parasite burden. Based on our findings, we propose a model in which prior non-lethal *Plasmodium* exposure primes macrophage-NK cell crosstalk. This interaction drives balanced cytokine production, enabling effective parasite control while limiting immunopathology (**Figure 9**).

While this study provides strong evidence for innate cross-protection, several limitations remain that offer directions for future research. The inherent plasticity of macrophages enables their functional polarization during infection, a process that critically influences disease resolution or progression (95). This is demonstrated in apicomplexan infections; for instance, *T. gondii* strains directly drive distinct macrophage polarization profiles (140). Having established a protective role for macrophages in our model of heterologous immunity, the next essential step is to define the specific subsets involved. To this end, we propose flow cytometric analysis of macrophage surface markers (CD86, CD206), intracellular staining for iNOS and Arg1 to determine polarization states (M1/M2) (141). Additionally, incorporating *ex vivo* phagocytosis assays with fluorescently labeled infected red blood cells will allow for a direct assessment of macrophage effector function. These approaches would be important for characterizing the functional phenotype of macrophages mediating protection in our co-infection model. Beyond phenotypic characterization, investigating immune cell dynamics at later time points (e.g., day 100 post-infection) is necessary to understand the loss of protection. This may be associated with a decline in activated macrophage and NK cell populations or reflects the transient nature of trained immunity, including epigenetic reprogramming. IL-17 links IFN-γ-mediated immunity with splenic organization and stress erythropoiesis during malaria (142–144). Although not assessed here, its contribution to the preserved splenic architecture and protection from severe anemia in co-infected mice warrants further investigation (145, 146). Finally, deciphering the roles of tissue-resident macrophages, particularly in the spleen, and their contribution to systemic cytokine homeostasis will be crucial. Such investigations would advance the understanding of the heterologous immunity described in our study and facilitate the translation of macrophage-driven innate protection into effective therapeutic strategies.

## Conclusions

Our previous work demonstrated that *Babesia* protects against *Plasmodium* (29). This study fills the reciprocal protection gap by showing that primary *Plasmodium* infection also confers cross-protection against lethal *Babesia*. Here, we identify the critical role of macrophages in providing cross-protection. A precise balance between pro- and anti-inflammatory responses governs this cross-protective immunity. Specifically, the coordinated production of IL-12p70, IFN-γ, TNF-α, and IL-10 in co-infected mice enabled effective suppression of lethal parasitemia while mitigating immunopathology driven by excessive inflammation (**Figure 9**). These results suggest that macrophage-targeted immunomodulation using *Plasmodium* may provide cross-protection against babesiosis and potentially revolutionize therapeutic strategies in co-endemic regions. Future research is required to understand the *Babesia*-suppressing effects of *Plasmodium*, with the ultimate goal of developing novel therapeutic tools to control babesiosis.

## Data Availability

The original contributions presented in the study are included in the article/**Supplementary Material**. Further inquiries can be directed to the corresponding authors.
