# Supplementary material for: Immunity beyond borders: non-lethal Plasmodium confers cross-protection against lethal Babesia via macrophage activation
Source: Front Immunol. 2026 May 5;17:1805513. doi: 10.3389/fimmu.2026.1805513 (PMC13183540; doi:10.3389/fimmu.2026.1805513)
Supplement: Supplementary file 1 [file DataSheet1.docx]

Supplementary Material

**Immunity beyond borders: non-lethal *Plasmodium* confers cross-protection against lethal *Babesia* via macrophage activation**

**Iqra Zafar^1,2*^, Li Yongchang^1,3^, Daisuke Kondoh^4^, Shimaa Abd El-Salam El-Sayed^1,5^, Tanjila Hasan^1,6^, Tomoyo Taniguchi^7^, Li Hang^1,8^, Noboru Inoue^1^, Kentaro Kato^2*^, Xuenan Xuan ^1,9*^**

**Correspondence: Prof. Xuenan Xuan, Prof. Kentaro Kato, Assistant Prof. Iqra Zafar**

## Supplementary Table 1.1. Antibody panel 1 used for FACS analyses

| **Immune cells** | **Anti-mouse antibody markers** | **Fluorophore** | **Antibody dilution (vol/vol μL)** | **Manufacturer (catalog no.)** |
| --- | --- | --- | --- | --- |
| Total leukocytes | CD45 | FITC | 0.5/200 | BioLegend (147709) |
| T lymphocytes | CD3 | PE/Cyanine7 | 1/200 | BioLegend (100219) |
| B lymphocytes | CD19 | Brilliant Violet 421^™^ | 1/200 | BioLegend (115537) |
| Natural killer cells | CD49b | PerCP/Cyanine5.5 | 1/200 | BioLegend (108915) |
| CD4 cells  CD8 cells | CD4  CD8a | PE  Brilliant Violet 510^™^ | 1/200  1/200 | BioLegend (100408)  BioLegend (100751) |

| **Immune cells** | **Anti-mouse antibody markers** | **Fluorophore** | **Antibody dilution (vol/vol μL)** | **Manufacturer (catalog no.)** |
| --- | --- | --- | --- | --- |
| Total leukocytes | CD45 | FITC | 0.5/200 | BioLegend (147709) |
| Macrophages | F4/80 | PE | 1/200 | BioLegend (123109) |
| Dendritic cells | CD11c | Brilliant Violet 510 ^™^ | 1/200 | BioLegend (117337) |

## Supplementary Table 1.2. Antibody panel 2 used for FACS analyses
